# Antigenic Peptide Prediction From E6 and E7 Oncoproteins of HPV Types 16 and 18 for Therapeutic Vaccine Design Using Immunoinformatics and MD Simulation Analysis

**DOI:** 10.3389/fimmu.2018.03000

**Published:** 2018-12-19

**Authors:** Basit Jabbar, Shazia Rafique, Outi M. H. Salo-Ahen, Amjad Ali, Mobeen Munir, Muhammad Idrees, Muhammad Usman Mirza, Michiel Vanmeert, Syed Zawar Shah, Iqra Jabbar, Muhammad Adeel Rana

**Affiliations:** ^1^Centre of Excellence in Molecular Biology, University of the Punjab, Lahore, Pakistan; ^2^Structural Bioinformatics Laboratory, Faculty of Science and Engineering, Biochemistry, Åbo Akademi University, Turku, Finland; ^3^Pharmaceutical Sciences Laboratory, Faculty of Science and Engineering, Pharmacy, Åbo Akademi University, Turku, Finland; ^4^Department of Genetics, Hazara University, Mansehra, Pakistan; ^5^Division of Science and Technology, University of Education Lahore, Lahore, Pakistan; ^6^Hazara University, Mansehra, Pakistan; ^7^Department of Pharmaceutical and Pharmacological Sciences, Rega Institute for Medical Research, Medicinal Chemistry, University of Leuven, Leuven, Belgium; ^8^School of Biological Sciences, University of the Punjab, Lahore, Pakistan; ^9^Department of Microbiology Quaid-i-Azam University, Islamabad, Pakistan

**Keywords:** binding affinity, computational, epitope prediction, docking, peptide vaccine, human papillomavirus, MHC-I

## Abstract

Human papillomavirus (HPV) induced cervical cancer is the second most common cause of death, after breast cancer, in females. Three prophylactic vaccines by Merck Sharp & Dohme (MSD) and GlaxoSmithKline (GSK) have been confirmed to prevent high-risk HPV strains but these vaccines have been shown to be effective only in girls who have not been exposed to HPV previously. The constitutively expressed HPV oncoproteins E6 and E7 are usually used as target antigens for HPV therapeutic vaccines. These early (E) proteins are involved, for example, in maintaining the malignant phenotype of the cells. In this study, we predicted antigenic peptides of HPV types 16 and 18, encoded by E6 and E7 genes, using an immunoinformatics approach. To further evaluate the immunogenic potential of the predicted peptides, we studied their ability to bind to class I major histocompatibility complex (MHC-I) molecules in a computational docking study that was supported by molecular dynamics (MD) simulations and estimation of the free energies of binding of the peptides at the MHC-I binding cleft. Some of the predicted peptides exhibited comparable binding free energies and/or pattern of binding to experimentally verified MHC-I-binding epitopes that we used as references in MD simulations. Such peptides with good predicted affinity may serve as candidate epitopes for the development of therapeutic HPV peptide vaccines.

## Introduction

Cervical cancer is the second most common cause of death in women worldwide after breast cancer (1). Strong molecular epidemiological evidence shows that persistent infection with high-risk human papillomavirus (HPV) is the major cause of invasive cervical cancer including condylomata (genital warts) and cervical dysplasia (2). HPV DNA is detected in more than 99% of all tumors of the uterine cervix. Of more than 40 HPV types that are transmissible through the genital area, types HPV16, HPV18, HPV31, HPV33, HPV35, HPV39, HPV45, HPV51, HPV52, HPV56, HPV58, and HPV59 belong to the group of high-risk type viruses by their malignant properties (3). From this group, HPV16 and HPV18 are together responsible for 70% of all cervical cancers (4, 5), HPV16, for example, causes ~46–63% of squamous cell carcinomas in the cervix worldwide (6) and is the most prevalent HPV type (55.1%) in invasive cervical cancer, HPV18 being the second most prevalent (14.3%) (5). Therefore, HPV types 16 and 18 are the principle targets for vaccine development. Three prophylactic HPV vaccines are available for HPV: (7) Cervarix, a bivalent HPV16/18 vaccine; Gardasil, a quadrivalent HPV16/18/6/11 vaccine; and Gardasil-9, an improved nonavalent version of Gardasil that is effective against a broader group of HPV types (HPV16/18/31/33/45/52/58/30/40) and, thus, should be more effective for example in Asian female population (8). These are virus-like particles based vaccines and have been confirmed to prevent most high-risk HPV infections and to minimize the consequences of HPV-associated diseases (9, 10). However, these vaccines have been shown to be effective only in individuals who have not been previously exposed to HPV. Moreover, the high prices of these vaccines have limited the use especially in low-income countries (4).

In addition to the prophylactic HPV vaccines, different types of therapeutic vaccines (live bacterial/viral vectors, RNA/DNA, protein/peptide and cell-based vaccines) are being developed and tested for the treatment of HPV-associated diseases [for recent reviews, see for example (11, 12)]. This study focuses on the peptide-based vaccine design. Peptide vaccines in general are considered to be safe, stable, and easy to manufacture (12). Most recent studies have focused on therapeutic vaccines against HPV16. For example, ISA Pharmaceuticals B.V. has a HPV16 peptide vaccine (ISA101) based on the Synthetic Long Peptide concept (SLP®) in clinical trials against HPV16 induced cervical cancer and other malignancies (13–16) (see isa-pharma.com). The main target antigens used for HPV therapeutic vaccines are the HPV early (E) proteins E6 and E7 that are constitutively expressed in HPV-associated cells (17). These oncoproteins are required for the generation and maintenance of the malignant cell phenotype (18). Their associated immune responses have been well characterized (19–21).

Unlike traditional prophylactic vaccines, therapeutic vaccines aim at principally activating the cell-mediated immune response. In general, the adaptive immunity is essential for combatting viral infections. However, especially the cellular immune response by cytotoxic T lymphocytes (CTLs) is crucial for protecting against many viruses, including HPV (22, 23) If the humoral response by the B-cell secreted antibodies fails to inhibit the virus particles from entering the cells, the infected cell starts producing the viral proteins. Some of these freshly synthesized proteins are degraded into fragments in the cell. Those peptide fragments that can bind to class I major histocompatibility complex (MHC-I) molecules are then transported to the cell surface. These peptide-MHC-I complexes are presented to an activated CD8^+^cytotoxic T cell that recognizes the complex with its T-cell receptor and lyses the infected cell by releasing cytotoxic substances. In addition, activated CD4^+^ T helper cells are needed for co-stimulating the proper activation of cytotoxic CD8^+^ T cells (24) CD4^+^ T cells are activated upon recognition of peptides that are presented to them by MHC class II (MHC-II) proteins on antigen presenting cells. Unfortunately, antibody-inducing traditional virus vaccines are not effective in activating T-cell responses. With peptide vaccines, however, it is possible to induce specific T-cell responses by including in a vaccine such peptide antigens that can be presented both by class I and II MHC molecules to T cells (24).

Consequently, the principle underlying the design of a therapeutic peptide vaccine is the identification of immunodominant B-cell and especially T-cell epitopes that have the ability to elicit specific immune responses (25). Even though the B-cell responses cannot clear out the intracellular viral oncoproteins in established infections, virus-specific antibodies might still be useful in inhibiting the entry of the viral proteins to new cells. Immunoinformatics (a branch of bioinformatics that uses computational approaches for understanding immunological data) has in the recent years made it easier to locate B-cell and T-cell epitopes in proteins and this in particular facilitates the identification of antigenic epitopes that are capable of stimulating an immune response (26, 27). This approach is cost-effective and convenient as the *in silico* predictions can reduce the number of experimentations needed (28). In the present study, an immunoinformatics approach was implemented for predicting and evaluating B-cell and T-cell antigenic sites of E6 and E7 proteins of HPV types 16 and 18 followed by a docking analysis with common MHC-I HLA molecules, with the aim to discover candidate peptides for the development of therapeutic vaccines against HPV types 16 and 18. The docked complexes were further analyzed by MD simulations to refine the docking poses and to evaluate the quality and strength of the binding interaction of the peptides with the MHC-I HLA molecules. Finally, we calculated the free energies of binding for the docked HPV peptides and representative crystallized peptides in complex with the studied MHC-I molecules to be able to predict the strongest binding peptides as candidates for peptide vaccine development.

## Materials and Methods

### Protein Structure Modeling and Validation

Since there are no available experimental structures of the HPV (types 16 and 18) E6 and E7 proteins, we built homology models for these proteins using the intensive mode of Phyre2 server (29). The server generates a full-length three-dimensional (3D) model of a protein sequence by employing both multiple template modeling and simplified *ab initio* folding simulation. The HPV protein sequences were obtained via the National Center for Biotechnology Information (NCBI) protein server (ncbi.nlm.nih.gov/protein): ACS92644.1 (E6 protein of HPV16), ACS92645.1 (E7 protein of HPV16) ALA62638.1 (E6 protein of HPV18) and NP_040311.1 (E7 protein of HPV18). Stereochemical quality, based on the distribution of dihedral angles of the modeled structures was evaluated by the Ramachandran plot (30). ModRefiner algorithm (31) was used for the refinement of the dihedral angle conformations in models with residues in unfavored regions of the Ramachandran plot. The 3D models were then utilized in the conformational (discontinuous) B-cell epitope predictions while the protein sequences were used for linear B-cell and T-cell epitope predictions.

### Surface Accessibility, Flexibility and Hydrophilicity Prediction

Emini Surface Accessibility Prediction was implemented for computing the surface accessibility (32) of the E6 and E7 protein residues. For predicting the residue flexibility, Karplus & Schulz flexibility prediction method (33) was used, and for obtaining a residue hydrophilicity profile, Parker hydrophilicity prediction (34) was applied. All the used tools were accessed from the IEDB analysis resource website: tools.immuneepitope.org/bcell/.

### Linear and Conformational B-Cell Epitope Prediction

Kolaskar & Tongaonkar Antigenicity method (http://tools.immuneepitope.org/bcell/) was selected for prediction of linear B-cell epitopes (35). This semi-empirical method of linear epitope prediction has been reported to have a prediction accuracy of about 75% when tested on a dataset of 169 experimentally known antigenic determinants. The method is based on the physicochemical properties of the residues and their frequencies of existence in experimentally known epitopes. The peptides reaching or crossing the threshold (about 1.05) were construed as potential antigenic epitopes.

ElliPro (available at the IEDB analysis resource website: tools.immuneepitope.org/ellipro/) was utilized for predicting the conformational epitopes for B-cells (36). The tool associates a so-called Protrusion Index (PI) of residues in the predicted epitopes, approximates protein shape and clusters the neighboring residues depending on the PI. ElliPro has been shown to perform the best in predicting conformational epitopes when using a benchmark dataset of antibody-protein complexes (36). It was compared to six other tools that are used for conformational epitope prediction and had an AUC (“area under the ROC curve”) value of 0.732 for the best predictions of each protein (AUC here represents the dependency between true positive rate and false positive rate). In addition, the best prediction was ranked among the first three for more than 70% of proteins and never worse than the fifth.

### Cytotoxic T-Cell Epitope Prediction

Prediction of cytotoxic T lymphocyte (CTL) epitopes in the E6 and E7 proteins of HPV types 16 and 18 was performed using the NetCTL-1.2 server (37), accessible from: cbs.dtu.dk/services/NetCTL. Conventional antigen processing and presentation to CTLs involves C-terminal cleavage of peptides from intracellular proteins by the proteasome and subsequent transport of a subset of the peptides to endoplasmic reticulum (ER) by Transporter associated with Antigen Presentation (TAP) molecules. In ER, peptides with correct size and suitable sequence motifs bind to MHC-I molecules and are moved to the cell surface, where CTLs recognize the complexes (37, 38) As an output, NetCTL server gives peptide sequences together with their predicted MHC-I binding affinity, binding affinity rescale value (normalized by the first percentile score), C-terminal cleavage affinity, and transport efficiency by TAP molecules. The server also computes an overall predicted score, which has a threshold of 0.75; hence, the peptide fragments corresponding to the prediction score >0.75 were predicted as potential CTL epitopes. NetCTL can predict antigenic epitopes that bind to 12 recognized supertypes of MHC-I HLA molecules (39) and thus, we evaluated all four HPV proteins against all the available MHC-I supertypes. In a large-scale benchmark study that used a dataset of known HIV epitopes (37), NetCTL-1.2 has been shown to have a sensitivity (true positive rate) of over 0.72 among the 5% top-scoring peptides, outperforming the other studied CTL epitope prediction methods.

### Biased Peptide Modeling and Flexible Docking

The 3D structures of the predicted antigenic peptides were modeled by a biased modeling method of the PEP-FOLD3 server (40). The peptide sequences were uploaded for modeling one by one. Simulations were set to 200 and models were sorted with the coarse-grained protein force field sOPEP (optimized potential for efficient structure prediction) (41, 42) and full conformational flexibility was allowed for the whole peptide sequence. As MHC-I receptors, we used the crystal structures of HLA alleles that were selected based on the antigenic epitope predictions (i.e., HLA-A^*^24:02—Protein Data Bank code: 2BCK; HLA-A^*^01:01—PDB code: 1W72; HLA-A^*^02:01–PDB code: 3HLA; HLA-B^*^44:02—PDB code: 3L3D). The binding motif residues belonging to the respective HLA allele were located from MHC Motif Viewer (43) to define the interaction patch that was given as input in PEP-FOLD3. From the repertoire of the predicted peptide-receptor complexes, we selected the ones giving the correct peptide orientation (C-terminal near the more flexible F pocket of the receptor) and good sOPEPscores. In addition, more extended rather than helical conformations of the peptide were chosen as the peptides binding to the MHC-I binding pocket exhibit more extended conformations than bent structures according to the multiple experimental peptide-MHC-I complexes present in the PDB.

The peptide-MHC complexes generated by PEPFOLD3 were further refined by the FlexPepDock server (44). It implements the Rosetta FlexPepDock protocol for high-resolution docking of flexible peptides using pre-optimization and high-resolution refinement steps for generating refined peptide-protein complexes from the input model complex. The number of low and high resolution runs were both set to 100 when submitting the complex for the refinement. Hydrogen bond interactions and possible steric clashes in the docked peptide-MHC complexes were analyzed with BIOVIA Discovery Studio (version 4.5; Accelrys Inc.). In addition, PyMOL (Schrödinger, LLC) was used for visualizing and analyzing the models.

### Molecular Dynamics Simulations

All the docked complexes as well as original crystal complexes of the studied MHC-I HLA proteins (HLA-A^*^24:02 – PDB ID: 2BCK; HLA-A^*^02:01 – PDB ID: 5HHP; HLA-A^*^01:01 – PDB ID: 1W72; HLA-B^*^44:02: PDB ID: 3L3D) were submitted to molecular dynamics (MD) simulations using the AMBER16 simulation package (45). After minimizing and equilibrating the solvated system [with TIP3P (46) water and Na^+^ as neutralizing counter ions], the production simulation was run at 300 K and at 1 bar pressure. We used the same overall simulation protocol as described in our previous study (47), but the length of the production run was increased from 5 to 10 ns. The cpptraj module of AMBER16 was used to analyze the trajectories. Prime-Molecular Mechanics Generalized Born Surface Area Prime-MMGBSA (48) module of Maestro (version 11.0.015; Schrödinger, Inc.) was used to estimate the free energy of binding of the peptides both in the initial (crystal or docked) complex structure as well as the final minimized structure from the MD simulations. The Prime-MMGBSA free energy of binding for the final complex was calculated both using a rigid complex as well as treating the protein residues within 4 Å from the peptide ligand as flexible. In addition, these calculated binding free energy values were compared with the binding affinity and ligand likelihood predictions for the studied peptide-MHC complexes by the NetMHCpan 4.0 server (49). This recently updated server was shown to identify the majority of natural ligands in the Pearson dataset (15,965 ligands and 27 HLA molecules) at a specificity of 98.5% using a percentile rank threshold of 2%. Hydrogen bonding in the final optimized complexes was also examined with BIOVIA Discovery studio and PyMOL.

## Results

### Homology Modeling and Structural Quality

**Figure 1:**
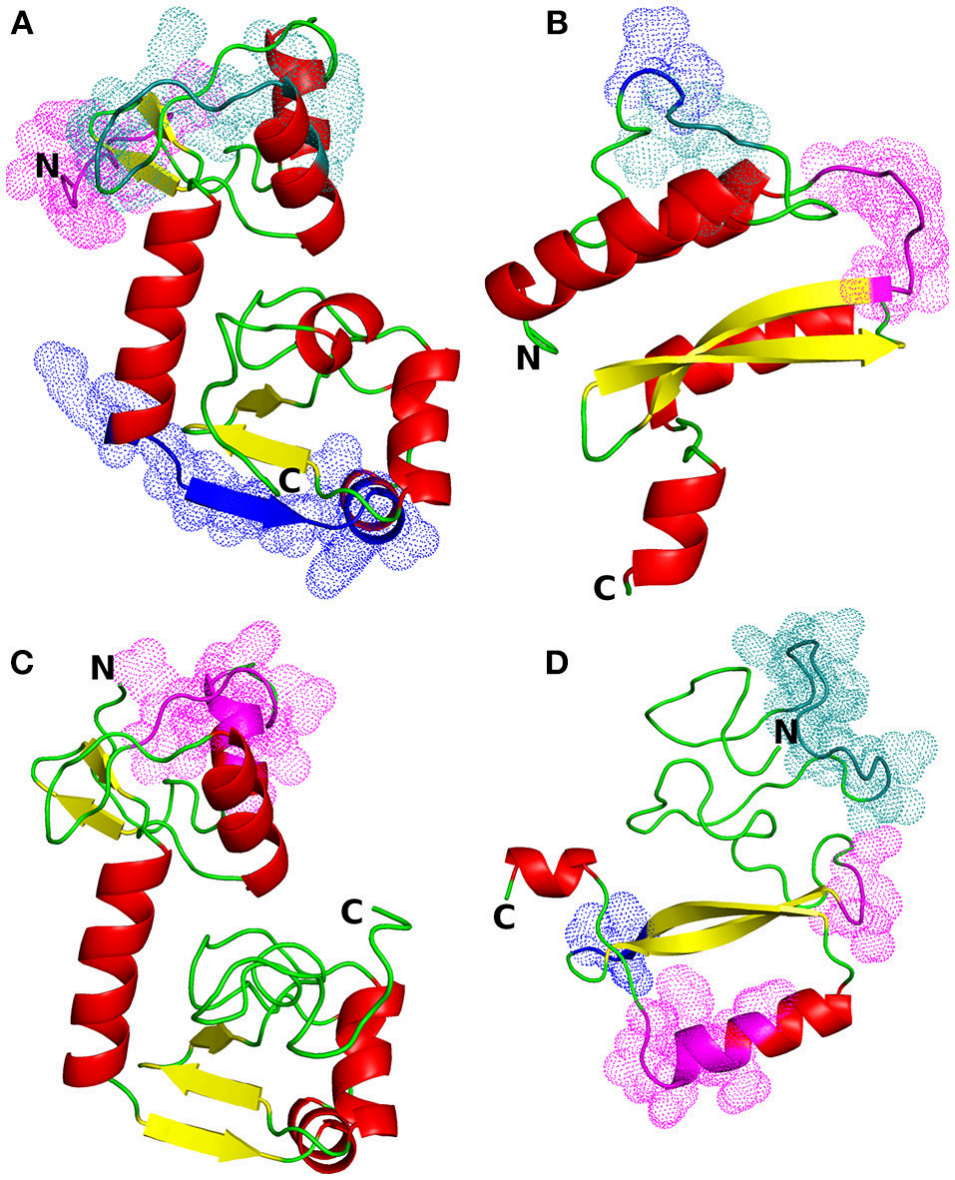
Structural models of HPV proteins (cartoon representation). The predicted conformational B-cell epitopes that coincide with the predicted CTL epitopes are shown as colored dots (cf. Tables 3, 4). **(A)** E6 protein of HPV type 16; magenta: residues 1–9; blue: 84–95; deep teal: 38, 40–46, 50. **(B)** E7 protein of HPV16; magenta: residues 45–51; blue: 26–27; deep teal: 23–25. **(C)** E6 protein of HPV18; magenta: residues 16, 18–21, 25–30. **(D)** E7 protein of HPV18; magenta: residues 55–57, 86, 88–89, 91–94; blue: 68–70; deep teal: 15–27.

The most suitable templates for the proteins of interest were identified to be the following PDB entries: 4XR8, chain F [crystal structure of HPV16 E6 mutant in complex with E6AP and p53; 2.25 Å resolution (50)] for E6 protein of HPV type 16 (identity: 97%, coverage: 95%), 2EWL [NMR structure of the C-terminal domain of the HPV45 E7, residues 55–106 (51)] for E7 protein of HPV type 16 (identity: 47%, coverage: 52%), 4GIZ [crystal structure of HPV16 E6 in complex with LXXLL peptide of E6AP; 2.55 Å resolution (52)] for E6 protein of HPV type 18 (identity: 59%, coverage: 87%) and 2EWL for E7 protein of HPV type 18 (identity: 77%, coverage: 49%) (see Figure 1 for the homology models and Figure S1 for the template-target pairwise sequence alignments). Since the template coverage for the E7 proteins was so low, the whole N-terminal side of the proteins (residues 1–42 and 1–49 for E7 HPV16 and HPV18, respectively) was modeled *ab initio* and, thus, is not so reliable. This can be seen as the completely different N-terminal regions in the E7 models; the E7 of HPV16 has a more structured N-terminus while E7 of HPV18 has a long unstructured N-terminal sequence stretch (Figures 1B,D). On the other hand, the templates for the E6 proteins covered most of the protein sequences, leaving just a short N-terminal stretch (from 3 to 7 residues for E6 of HPV16 and HPV18, respectively), and for E6 of HPV18 the C-terminus (13 residues) to be modeled without a structural template (Figure S1).

Ramachandran plot of the modeled E6 protein of HPV type 16 showed 98.1% residues in the favored and allowed regions while only 1.9% residues were in the outlier region, indicating the suitability of the structure for further analysis. The homology model of the E7 protein of HPV type 16 had 82.3% residues in favored and allowed regions. After the refinement of the model with the ModRefiner algorithm, 99% of the model residues were in the favored and allowed regions of the plot. Ramachandran plots of the modeled E6 and E7 proteins of HPV type 18 showed 99.4 and 95.1% of residues in the favored and allowed regions, respectively Figure S2; see also Figure S3 for the Ramachandran plots of the templates used).

### Surface Accessibility, Flexibility, and Hydrophilicity Prediction

Emini Surface Accessibility Prediction was implemented for computing the surface accessibility of residues based on the sequence of the viral proteins. For an antibody to recognize and bind to the antigen, the antigenic site must be surface accessible, i.e., exposed on the surface of the protein molecule. Also, the exposed areas come into contact with the hydrophilic environment so the potential antigenic sites are also hydrophilic. Parker hydrophilicity prediction tool was used for predicting the hydrophilic sites on the surface of the proteins. Karplus & Schulz flexibility prediction guides toward resolving potential linear antigenic sites as these segments of protein chain tend to be highly flexible (33). Flexibility allows the formation of an antigen-antibody interface since flexible regions can adjust their conformation upon interaction with an antibody; hence flexibility of a protein region is an indicator of the existence of a potential antigenic site (53). The top predictions for each protein are shown in Table 1 while all the predictions as graphs are available in Figures S4–S6. For the E7 proteins, the sites with the highest prediction values of all three parameters (surface accessibility, flexibility, and hydrophilicity) concentrate unanimously on a highly polar and negatively charged region along the N-terminus, whereas for the E6 proteins, the predicted, highly polar, and charged protein segments contain also basic residues and show a consensus site at the C-terminus. From the homology models of the HPV proteins one can also see that the protein segments predicted most accessible, hydrophilic, and flexible match mostly the unstructured termini or loop areas. Only the E6 protein of HPV16 residues 119–125 (PEEKQRH) predicted to be the most flexible, are located in a short alpha helical segment in the protein model.

**Table 1:** Predicted sequence stretches that are the most surface accessible, flexible, and hydrophilic in E6 and E7 proteins belonging to HPV types 16 and 18.

**Prediction type**	**Start**	**End**	**Sequence**	**Prediction score**
**E6 PROTEIN OF HPV TYPE 16**				
Surface accessibility	13	18	QERPRK	5.262
Flexibility	119	125	PEEKQRH	1.089
Hydrophilicity	149	155	SSRTRRE	5.514
**E7 PROTEIN OF HPV TYPE 16**				
Surface accessibility	32	37	SEEEDE	4.333
Flexibility	29	35	NDSSEEE	1.114
Hydrophilicity	30	36	DSSEEED	8.057
**E6 PROTEIN OF HPV TYPE 18**				
Surface accessibility	151	157	QRRRETQ	5.425
Flexibility	144	150	RARQERL	1.072
Hydrophilicity	151	157	QRRRETQ	5.371
**E7 PROTEIN OF HPV TYPE 18**				
Surface accessibility	35	40	EEENDE	5.166
Flexibility	32	39	SDSEEEND	1.099
Hydrophilicity	33	39	DSEEEND	8.129

### Linear and Conformational B-Cell Epitope Prediction

The linear B-cell epitopes were predicted with the Kolaskar & Tongaonkar method that detects antigenic determinants on the basis of hydrophobic residues (e.g., Cys, Leu, and Val) on the surface of a protein. The predicted linear B-cell epitopes of all proteins are presented in Table 2 (see also the graphical representations of the predicted epitopes in Figure S7). The conformational B-cell epitopes were predicted from the HPV protein models using ElliPro. The highest probabilities for conformational B-cell epitopes in E6 and E7 proteins of HPV type 16 were computed as 79.9% (PI score: 0.799) and 80.8% (PI score: 0.808), respectively. Moreover, the highest probabilities for conformational B-cell epitopes in E6 and E7 proteins of HPV type 18 were computed to be 77.8 and 72.4% (PI score: 0.778 and 0.724), respectively. Importantly, the predicted conformational B-cell epitopes of E7 proteins that have a PI score higher than 0.609 are located in the more reliably modeled regions of the protein structures. Amino acid residues, the number of residues, sequence location as well as the PI scores of the predicted conformational epitopes are given in Table 3 and the graphical depiction of these epitopes can be seen in Figure S8. Especially the conformational epitopes coincide with the top-predicted protein segments for surface accessibility, flexibility, and hydrophilicity. From a total number of 24 predicted linear and 19 conformational B-cell epitopes of the four HPV proteins, many epitopes were also predicted to be CTL epitopes (see Table 4 and Figure 1).

**Table 2:** Predicted linear B-cell epitopes of E6 and E7 proteins of HPV types 16 and 18.

**Start**	**End**	**Peptide^**a**^**	**Length**
**E6 PROTEIN OF HPV TYPE 16**			
19	25	LPQLCTE	7
32	43	DIILEC**VYCKQQ**	12
55	61	**RDLCI**VY	7
67	77	**YAVCDKCL**KFY	11
84	90	**RHYCYSL**	7
101	120	KPLCDLLIRCINCQKPLCPE	20
144	149	MSCCRS	6
**E7 PROTEIN OF HPV TYPE 16**			
21	27	**DLYCYEQ**	7
52	62	**Y**NIVTFCCKCD	11
64	73	TLRLCVQSTH	10
73	79	HVDIRTL	7
88	95	GIVCPICS	8
**E6 PROTEIN OF HPV TYPE 18**			
14	18	**LPDLC**	5
29	47	**EITCV**YC**KTVLELTEVV**EF	19
50	58	**KDLFVV**YRD	9
62	71	HAACHKCIDF	10
99	109	YNLLIRCLRCQ	11
136	144	GQCHSCCNR	9
**E7 PROTEIN OF HPV TYPE 18**			
9	15	QDIVLH**L**	7
23	30	**VDLLC**HEQ	8
62	69	**L**CMCCK**CE**	8
71	78	**RIELVV**ES	8
86	89	**FQQL**	4
94	102	**L**SFVCPWCA	9

a *residues highlighted in bold were also predicted to be CTL epitopes*.

**Table 3:** ElliPro predicted conformational B-cell epitopes of E6 and E7 proteins of HPV types 16 and 18.

**Prediction**	**Residues^**a**^**	**Number of residues**	**PI score^**b**^**
**E6 PROTEIN OF HPV TYPE 16**			
1	**M1**, **H2**, **Q3**, **K4**, **R5**, **T6**, **A7**, **M8**, **F9**, Q10, D11, P12, Q13, E14, R15, D63, G64, N65	18	0.799
2	Q123, L126, D127, K128, K129, Q130	6	0.694
3	I80, **R84**, **H85**, **Y86**, **C87**, **Y88**, **S89**, **L90**, **Y91**, **G92**, **T93**, **T94**, **L95**, E96, Q97, Q98, Y99, N100, K101, P102, C104, D105, L106, C118, P119, E120, R131, R136	28	0.677
4	R148, S149, S150, R151, T152, R153, R154	7	0.649
5	E155, T156, Q157, L158	4	0.649
6	C23, T24, E25, L26, Q27, T28, T29, I30, H31, D32, I33, I34, L35, E36, C37, **V38**, **C40**, **K41**, **Q42**, **Q43**, **L44**, **L45**, **R46**, R47, **Y50**	25	0.623
**E7 PROTEIN OF HPV TYPE 16**			
1	V90, C91, P92, I93, C94, S95, Q96, K97, P98	9	0.808
2	**A45**, **E46**, **P47**, **D48**, **R49**, **A50**, **H51**, Q70, S71, T72, H73, V74, D75, T78, L82, G85, T86, L87	18	0.719
3	**E26**, **Q27**, L28, N29, D30, S31, S32, E34, E35, D36	10	0.609
4	H2, G3, D4, T5, P6	5	0.586
5	**Y23**, **C24**, **Y25**	3	0.507
**E6 PROTEIN OF HPV TYPE 18**			
1	C142, N143, A145, R146, Q147, E148, R149, L150, Q151, R153, R154, E155, T156, Q157, V158	15	0.778
2	I75, L78, R79, H80	4	0.733
3	Y81, S82, D83, S84, V85, Y86, G87, D88, T89, L90, E91, K92, L93, T94, N95, T96, G97, L98, Y99, N100, L101, R126	22	0.716
4	M1, A2, R3, F4, E5, D6, P7, T8, R9, R10, P11, Y12, **D16**, **C18**, **T19**, **E20**, **L21**, N22, T23, S24, **L25**, **Q26**, **D27**, **I28**, **E29**, **I30**, D58, S59, I60	29	0.71
5	L118, L121, N122, E123, K124, R125	6	0.552
**E7 PROTEIN OF HPV TYPE 18**			
1	**E55**, **P56**, **Q57**, S78, S79, A80, D81, D82, R84, A85, **F86**, **Q88**, **L89**, **L91**, **N92**, **T93**, **L94**, S95	18	0.724
2	C65, C66, K67, **C68**, **E69**, **A70**, C98, P99, W100, C101, A102, S103, Q104, Q105	14	0.706
3	M1, H2, G3, P4, K5, A6, T7, L8, I11, L13, H14, **L15**, **E16**, **P17**, **Q18**, **N19**, **E20**, **I21**, **P22**, **V23**, **D24**, **L25**, **L26**, **C27**, E40	25	0.594

a *residues highlighted in bold were also predicted to be CTL epitopes*;

b *Protrusion Index of ElliPro; a higher value indicates a higher probability for a discontinuous B-cell epitope*.

**Table 4:** NetCTL 1.2 prediction of CTL epitopes from the E6 and E7 proteins of HPV16 and 18.

**Sequence position**	**Peptide sequence^**a**^**	**Predicted MHC-I binding affinity**	**C-terminal cleavage affinity**	**Transport affinity**	**Prediction score^**b**^**
E6 PROTEIN PEPTIDES FROM HPV TYPE 16 BINDING TO MHC-I SUPERTYPE HLA-A*24					
87	**CYSLYGTTL**	0.4932	0.9759	1.122	1.2526
49	V**Y**DFAF**RDL**	0.4382	0.9716	0.938	1.1258
82	EY**RHYCYSL**	0.4006	0.9713	1.118	1.0546
51	DFAF**RDLCI**	0.43	0.1600	0.351	0.9571
66	P**YAVCDKCL**	0.3714	0.5291	0.897	0.9151
1	**MHQKRTAMF**	0.2304	0.9662	2.555	0.7632
38	**VYCKQQLLR**	0.2938	0.2405	1.813	0.7524
E7 PROTEIN PEPTIDES FROM HPV TYPE 16 BINDING TO MHC-I SUPERTYPE A*01					
44	Q**AEPDRAHY**	0.2226	0.8992	2.816	1.221
19	TT**DLYCYEQ**	0.2241	0.4958	−0.491	1.0014
15	LQPETT**DLY**	0.1587	0.9669	2.85	0.9614
E6 PROTEIN PEPTIDES FROM HPV TYPE 18 BINDING TO MHC-I SUPERTYPE A*02					
13	K**LPDLCTEL**	0.7424	0.9781	1.014	1.3041
47	FAF**KDLFVV**	0.7669	0.9546	0.217	1.2972
37	**TVLELTEVV**	0.5402	0.8306	0.551	0.9574
25	**LQDIEITCV**	0.4746	0.7534	0.272	0.8341
36	**KTVLELTEV**	0.4659	0.4919	0.471	0.7918
E7 PROTEIN PEPTIDES FROM HPV TYPE 18 BINDING TO MHC-I SUPERTYPE B*44					
54	A**EPQ**RHTM**L**	0.5238	0.9754	1.08	1.4984
15	**LEPQNEIPV**	0.4885	0.7506	0.145	1.3303
68	**CEARIELVV**	0.3716	0.8931	0.256	1.0677
86	**FQQL**F**LNTL**	0.3186	0.9319	1.057	0.9822
19	**NEIPVDLLC**	0.3065	0.0417	−0.064	0.7626

a *amino acids highlighted in bold were also predicted as B-cell antigenic sites (linear and/or conformational)*;

b *prediction score threshold >0.75000*.

### Cytotoxic T-Cell Epitope Prediction

Based on the NetCTL-1.2 epitope prediction results against different MHC-I supertypes, we selected the MHC-I supertype/HPV protein combinations that gave the best prediction values and CTL epitopes that also overlapped with the predicted B-cell epitopes. For E6 protein of HPV16, HLA-A^*^24 supertype was selected; for E7 protein of HPV16, HLA-A^*^01; for E6 protein of HPV18, HLA-A^*^02; and for E7 protein of HPV18, HLA-B^*^44. Table 4 represents the predicted epitope peptide sequences with their predicted MHC-I binding affinity, proteasomal C-terminal cleavage affinity, TAP transport efficiency, and also the overall predicted score, which has a threshold of 0.75 (the peptides with a prediction score >0.75 are hence predicted as potential CTL epitopes). All the predicted nonapeptidic CTL epitopes (from all the four proteins) that are presented in Table 4 coincided at least partially with the sites for the predicted linear and/or conformational B-cell epitopes and were selected for further analysis by docking. However, the N-terminal peptide MHQKRTAMF of E6 protein from HPV16 that had the lowest predicted binding affinity to MHC-I HLA-A^*^24 from that group of epitopes was left out from the further analysis.

### Biased Peptide Modeling and Flexible Docking

In the docking procedures and subsequent MD simulations of the HPV peptides, MHC I alleles HLA-A^*^2402, HLA-A^*^0101, HLA-A^*^0201, and HLA-B^*^4402 were used as receptor structures. HLA-A^*^2402 is one of the most common MHC-I types as it exhibits abundant expression in Caucasians and oriental populations (54, 55) HLA-A^*^0101 and HLA-A^*^0201 alleles have been reported to be among the few relatively high frequency alleles contributing to a greater percentage of HLA-A locus alleles. HLA-A^*^0201 represents frequency of 27.1% in Caucasians, 21.7% in North American Natives and 23.1% in Hispanics while 12.3% in African Americans, and 9.47 % in Asians. HLA-A^*^0101 accounts for 15.09% in Caucasians, 7.49% in North American Natives, 5.98% in Hispanics, 5.56% in African Americans and 1.53% in Asians. Similarly, from the collection of HLA-B alleles, HLA-B^*^4402 was chosen as it is among highly frequent HLA-B alleles. Expression has been reported to be 11.7% in Caucasians, 4.28% in North American Natives, 3.42% in Hispanics, 1.99% in African Americans while only 0.7% in Asians (55).

**Figure 2:**
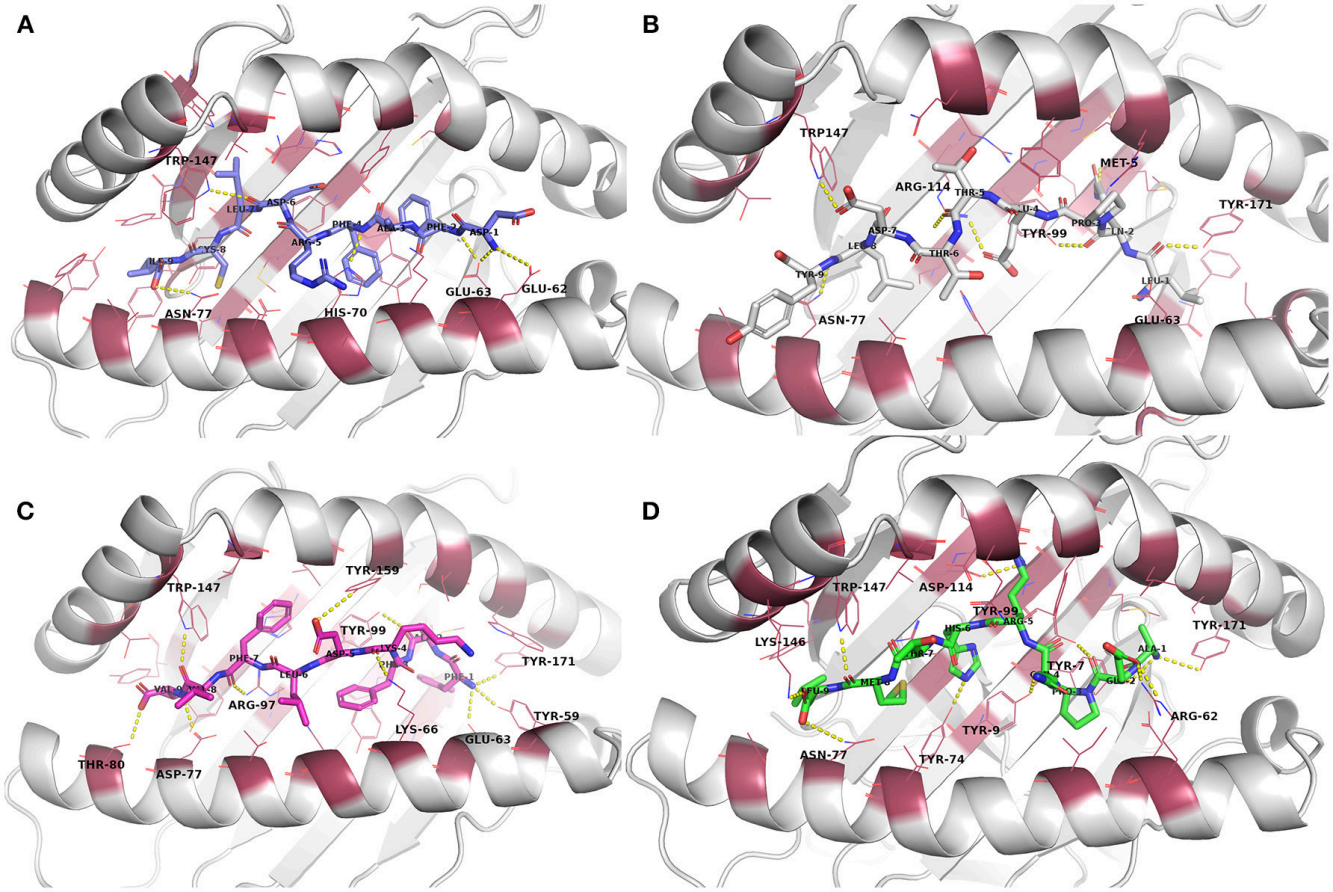
Docked epitope candidates (shown as sticks) at their MHC-I receptor binding sites (the receptor is shown as cartoon and the binding site residues within 4 Å from the peptide as raspberry red lines). Polar interactions are denoted with yellow dashed lines (detected with PyMOL v. 2.1.0, Schrödinger, LLC). Atom color code for non-carbon atoms: blue: nitrogen, red: oxygen; yellow: sulfur. Hydrogen atoms were omitted for clarity. **(A)** E6 (HPV type 16) peptide DFAFRDLCI (slate carbon atoms) at the binding pocket in the receptor HLA-A*24:02. **(B)** E7 (HPV type 16) peptide LQPETTDLY (white carbon atoms) at HLA-A*01:01. **(C)** E6 (HPV type 18) peptide FAFKDLFVV (magenta carbon atoms) at HLA-A*02:01. **(D)** E7 (HPV type 18) peptide AEPQRHTML (green carbon atoms) at HLA-B*44:02.

From the biased peptide modeling using PEPFOLD3, the structure giving correct orientation and lowest sOPEP values was selected for flexible refinement of the modeled peptide-MHC complexes. See Table S1 for the modeling/docking scores as well as H bond interactions in the complexes. No steric clashes were observed in the complexes after PEPFOLD3 modeling and FlexPepDock refinement. The modeled complexes before MD simulation are presented in Figure 2 and Figures S9–S12.

Evaluation of the docking results (Table S1) indicated that, for E6 protein (HPV16), the complexes with peptides EYRHYCYSL, PYAVCDKCL, and VYCKQQLLR had stronger H-bonding interactions according to their respective FlexPepDock H-bond energy of sidechain interactions in comparison to the other E6 protein (HPV16) peptides. Glu62 and Glu63 (atoms OE1 and OE2) belonging to HLA-A^*^24:02 were found to be interacting with the peptide sidechain atoms in most of the complexes. All of the E6 protein (HPV16) peptides retained some of the initial H-bonding interactions after the MD simulation except EYRHYCYSL.

From the E7 protein (HPV16) peptides docked to HLA-A^*^01:01, all three peptides (i.e., QAEPDRAHY, TTDLYCYEQ, and LQPETTDLY) exhibited reasonable side chain H-bonding energy values (< −20) but retained only few (or none) initial H-bonding interactions after the MD simulation. Arg114 side chain amino group of the MHC protein was found as a common hydrogen bond donor in all of the E7 protein (HPV16) peptide complexes.

With peptides of E6 protein (HPV18) docked to HLA-A^*^02:01, common hydrogen bond forming residues of the MHC protein in most of the complexes were Lys66 and Trp147. After the MD simulation, all of the E6 protein (HPV18) peptides retained some of initial H-bonding interactions except TVLELTEVV. However, all these peptides had less negative H-bonding side chain energy (>-20), indicating lesser binding strength with this particular MHC protein than the other docked peptide groups with their respective MHC-receptors used in this study.

Among the five E7 protein (HPV18) peptides docked to HLA-B^*^44:02, three of the peptides had a better FlexPepDock sidechain H-bonding strength (AEPQRHTML: −27.15, LEPQNEIPV: −23.68 and NEIPVDLLC: −23.89) than the others. All the peptides retained only few (or none) of the initial interactions after MD simulation except the peptide NEIPVDLLC that retained seven initial H-bonding contacts after the MD simulation.

### Molecular Dynamics Simulations

**Figure 3:**
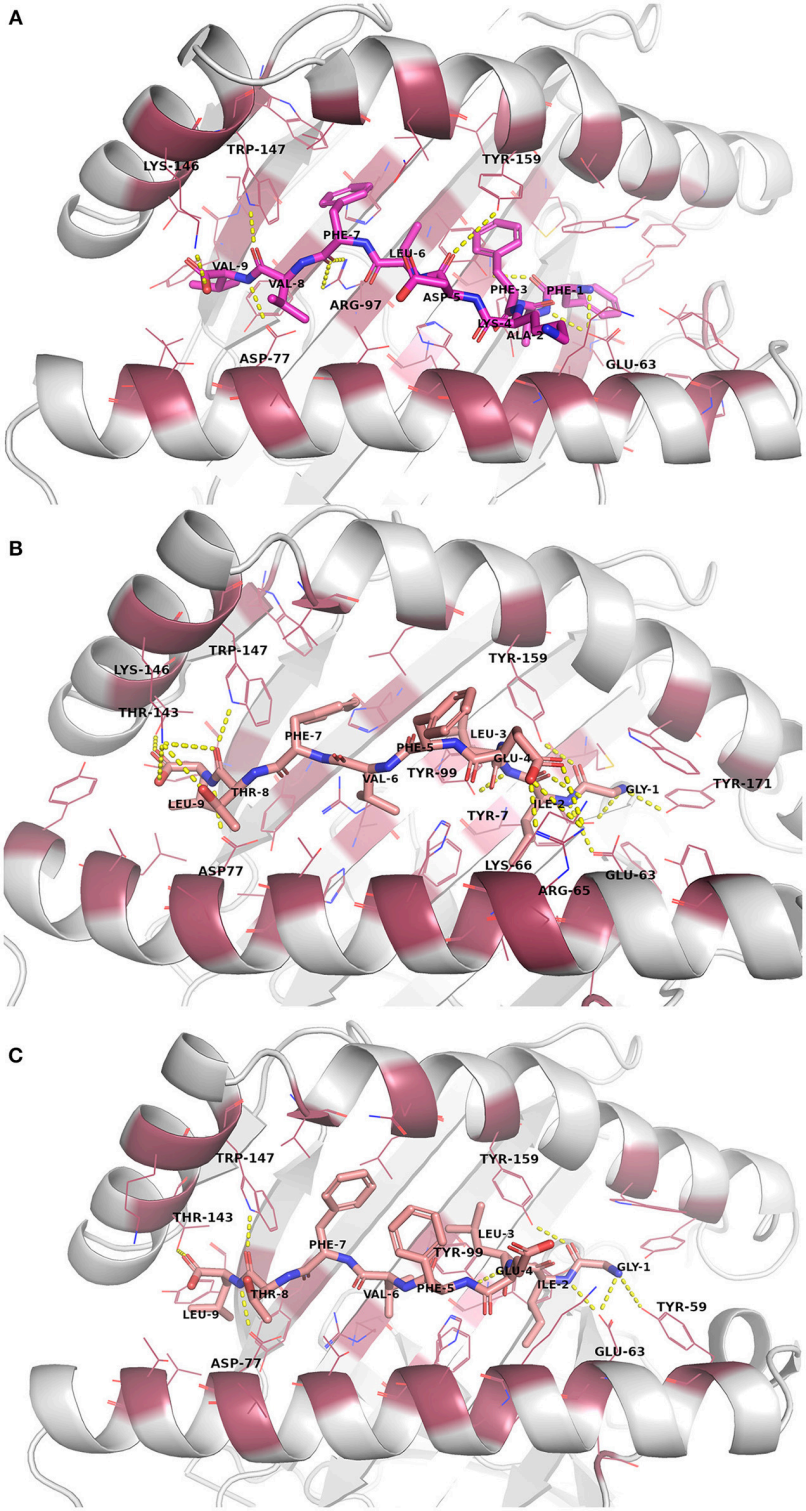
Binding site interactions after the molecular dynamics (MD) simulation (the receptor is shown as cartoon, the peptides as sticks, and the binding site residues within 4 Å from the peptide as raspberry red lines). Polar interactions are denoted with yellow dashed lines (detected with PyMOL v. 2.1.0, Schrödinger, LLC). Atom color code for non-carbon atoms: blue: nitrogen, red: oxygen; yellow: sulfur. Hydrogen atoms were omitted for clarity. **(A)** Peptide FAFKDLFVV (magenta carbon atoms) at its receptor HLA-A*02:01. **(B)** The reference crystal complex PDB ID: 5HHP (peptide GILEFVFTL [salmon carbon atoms] at HLA-A*02:01) before MD. **(C)** The reference crystal complex PDB ID: 5HHP after MD.

The docked peptide-MHC protein complexes were further refined and their stability was investigated by performing 10-ns molecular dynamics (MD) simulations of the complexes in an explicit water system at 300 K. In addition, experimentally determined peptide-MHC complexes of the studied MHC-I proteins were also simulated using the same protocol to compare their stability and binding pattern with that of the predicted epitope-MHC complexes. In general, the potential energy of all the simulation systems remained stable during the MD simulations (data not shown). Root-mean-square deviation (RMSD) of the crystal complexes was in general somewhat lower (ca. 1.5 Å for the MHC-I peptide-binding domain) than with the docked complexes, although only few complexes had values over 2.0 Å for the backbone atoms of the MHC-I peptide-binding domain (Figures S13–S16). RMSD values of the peptides in the complexes remained mostly between 1–1.5 Å (Figures S17–S20, Table 5). In the crystal complex PDB ID: 5HHP the co-crystallized peptide GILEFVFTL was the most stable with RMSD of only about 0.5 Å, and FAFKDLFVV, E6 protein peptide from HPV type 18 had a comparable RMSD in the binding groove of MHC-I HLA-A^*^02:01 (Figure 3 and Figure S18). These two peptides exhibited the best initial Prime-MMGBSA free energies of binding (Table 5). Most of the peptides adopted a better pose during the simulation, which is seen in the improved Prime-MMGBSA energy values. However, most of the experimental complexes improved the binding energy value only slightly if at all during the MD simulation. Treating the binding site flexible did not improve the Prime-MMGBSA energies for the experimental structures, although it did not worsen the values much either. For some docked peptides the flexible treatment improved the free energy of binding value (e.g., FAFKDLFVV) but mostly it worsened the values.

**Table 5:** Dynamics and energetics of the HPV peptide-MHC-I complexes.

**HPV peptide sequence**	**RMSD peptide (Å)^**a**^**	**Change in the MHC-I binding groove (F pocket) size**		**MM-GBSA dG (kcal/mol) before MD**	**MM-GBSA dG (kcal/mol) after MD (flex-4Å)^**c**^**	**NetMHCpan 4.0 prediction %Rank^**d**^**
		**d1/d2^**b**^ (Å) (initial)**	**d1/d2 (Å) (after MD)**			
E6 PROTEIN PEPTIDES FROM HPV TYPE 16 BOUND TO MHC-I HLA-A*24:02						
CYSLYGTTL	1.836	9.4/20.2	11.5/21.5	−27.51	−94.47 (−120.31)	*0.5277*/**0.3687**
DFAFRDLCI	1.949	9.5/20.0	10.4/21.6	−40.40	−137.25 (−133.01)	4.8315/4.5638
EYRHYCYSL	1.409	9.6/20.1	10.9/20.8	−25.47	−111.19 (−85.55)	*1.5430*/*0.6899*
PYAVCDKCL	1.290	9.6/20.5	15.1/21.4	−60.09	−127.72 (−123.47)	4.5376/4.0033
VYCKQQLLR	1.477	9.3/20.2	**8.4/19.3**^e^	−35.22	−64.54 (−75.49)	8.6743/11.4168
VYDFAFRDL	1.124	9.5/20.4	10.2/19.3	−65.68	−99.73 (−109.08)	**0.2996/***0.8693*
VYGFVRACL (PDB ID: 2BCK)	1.401	9.8/20.0	10.1/20.5	−110.41	−126.44 (−125.26)	*0.5940*/**0.2979**
E7 PROTEIN PEPTIDES FROM HPV TYPE 16 BOUND TO MHC-I HLA-A*01:01						
LQPETTDLY	1.957	9.0/20.6	11.4/19.1	23.68	−90.71 (−67.53)	*0.6525*/*1.5965*
QAEPDRAHY	1.239^f^	9.1/20.8	**8.2/19.9**^e^	9.72	−50.27 (−45.55)	**0.2306**/*1.0692*
TTDLYCYEQ	1.096^f^	9.1/20.5	12.0/22.3	−1.17	−51.87 (−34.05)	2.2119/*1.0697*
EADPTGHSY (PDB ID: 1W72)	1.413	9.2/20.6	12.2/20.5	−76.96	−124.38 (−118.93)	**0.0191/0.0950**
E6 PROTEIN PEPTIDES FROM HPV TYPE 18 BOUND TO MHC-I HLA-A*02:01						
FAFKDLFVV	1.998	9.4/20.5	11.7/21.7	−97.91	−145.26 (−152.00)	**0.4331**/**0.1569**
KLPDLCTEL	2.978	9.3/20.7	8.4/23.4	−38.53	−93.36 (−91.54)	**0.2375**/*0.6223*
KTVLELTEV	1.316	9.4/20.5	11.6/20.1	−51.25	−48.50 (−48.36)	*1.0250*/*1.9481*
LQDIEITCV	2.107	9.3/20.3	11.6/20.8	−32.12	−98.60 (−106.28)	3.2461/4.8162
TVLELTEVV	2.193	9.4/20.5	10.7/20.6	−70.66	−68.52 (−28.78)	*0.8711*/2.1996
GILEFVFTL (PDB ID: 5HHP)	0.718	10.0/20.3	10.7/21.6	−123.06	−117.36 (−123.66)	**0.0362**/**0.0430**
E7 PROTEIN PEPTIDES FROM HPV TYPE 18 BOUND TO MHC-I HLA-B*44:02						
AEPQRHTML	2.278	9.1/20.0	10.5/22.7	−76.43	−96.99 (−96.86)	*0.6938*/3.1601
CEARIELVV	1.183	9.1/20.0	15.8/18.5	−21.90	−62.25 (−65.96)	4.2873/*1.1049*
FQQLFLNTL	1.637	9.1/19.9	10.2/24.7	−59.83	−59.52 (−71.81)	10.2991/8.0843
LEPQNEIPV	1.441	9.1/20.0	11.2/23.6	−26.21	−75.08 (−77.78)	5.4620/8.7051
NEIPVDLLC	1.933	9.0/19.9	7.4/22.6	−42.70	−67.32 (−64.47)	2.2264/3.9074
EEAGRAFSF (PDB ID: 3L3D)	1.214	9.3/19.6	12.2/22.0	−91.15	−89.01 (−87.84)	**0.0341/0.0718**

a *RMSD of the Cα atoms between the initial docked peptide conformation and the minimized conformation from the final MD frame*;

b d1, distance between the Cα atoms of Tyr85 in α1 helix and Met138 in α2 helix; d2, distance between the Cα atoms of Asp74 in α1 helix and Ala149 in α2 helix (Tyr74 and Thr138 in HLA-B^*^44:02);

c *Flex-4Å, During the Prime/MM-GBSA calculation protein flexibility within 4 Å distance from the ligand was allowed*;

d *Eluted ligand likelihood prediction/binding affinity prediction (weak binders in italics, limit < 2%, strong binders in bold, limit < 0.5%)*;

e *F pocket size has been reduced during the MD simulations*;

f *only 8 atoms of 9 aligned*.

The root-mean-square fluctuations (RMSF) of the individual peptide residues showed that especially the residues in N-terminus (at position 2, P2) were generally tightly bound in the MHC-I groove whereas the residues in the middle (at P4–P6) seemed to be more loosely bound and could fluctuate around at their site (Figures S21–S24). The C-terminus of the crystal complex peptides was also tightly bound (RMSF 0.7–0.8 Å at P9) except for PDB ID: 3L3D (RMSF ca. 1.6 Å at P9) (Figure S16). Of note, the peptide ligand in the 3LD3 crystal is the F3A mutant of a high-affinity self-peptide derived from DPα^*^0201 (EEFGRAFSF). This mutation causes a significant change in the peptide conformation, possibly leading to diminished binding affinity and thus, compromised immunogenicity (56). The predicted peptides had somewhat larger RMSF values at P9 anchor position than the experimental peptides, with a few exceptions (ranging from ca. 0.75 Å of DFAFRDLCI at MHC-I HLA-A^*^24:02 to ca. 1.8 Å of QAEPDRAHY at MHC-I HLA-A^*^01:01).

Closing of the MHC-I binding groove can be observed from the reduced F pocket size. The F pocket binds the C-terminal residue of the nonapeptides. During the MD simulations the F pocket changed its size (57) variably depending on the peptide that was inside the groove (Table 5, Figures S25–S28). In most of the complexes, the size of the pocket enlarged somewhat, including the experimental complexes for which the greatest change was in the PDB ID: 3L3D complex.

The NetMHCpan 4.0 predicted binding affinities and ligand likelihoods were to some extent consistent with the calculated Prime-MMGBSA energies. Of note, all the experimental peptides were predicted as strong binders. Surprisingly, DFAFRDLCI, E6 peptide of HPV16 that had one of the best Prime-MMGBSA energies, was not predicted to be even a weak binder. That is likely due to phenylalanine in the place of tyrosine at P2 since mutating that residue to tyrosine improves the binding level prediction of the epitope to a weak binder (data not shown). On the other hand, FAFKDLFVV that showed the best Prime-MMGBSA energy was consistently predicted as a strong binder by the server.

## Discussion

Immunoinformatics has been shown to be useful in predicting antigenic peptide B-cell and T-cell epitopes for peptide vaccine development [for recent reviews see for example (58, 59)]. Studying the peptide-MHC interactions by molecular docking studies [see for example references (60–65)] has been used to aid in evaluating the binding affinity of the predicted peptide fragments since a sufficient binding affinity to an antigen presenting MHC-I protein is the most critical requirement for a peptide to elicite a proper CTL response (37, 66). In general, the docking method and results need to be validated against the available experimental peptide-MHC complex structures. However, it is also well known that the current docking methods have their limitations and cannot always generate docking poses similar to the experimentally verified binding modes. Docking flexible oligopeptides is even more challenging than docking small molecules. Thus, there is often a need to refine the docked complexes using MD simulations [for example references (47, 67, 68)] or other flexible refinement methods such as FlexPepDock (44). Moreover, completely new docking algorithms have been recently developed specifically for docking peptides to MHC molecules (60, 61). Also, a relatively straightforward way of building peptide-MHC complexes using modeling software is to mutate the residues in an experimental peptide complexed with the target MHC structure to those of the desired epitope sequence. However, this also requires a subsequent energy minimization and depending on the degree of dissimilarity of the modeled and the original peptide, also longer or shorter MD simulations to refine the complex.

The present study entails a combination of immunoinformatics, docking and MD simulation analysis for the evaluation of the binding affinity of candidate peptides of E6 and E7 proteins (belonging to HPV types 16 and 18). In addition to predicting the most promising peptide epitopes for HPV vaccine development, this study further develops our previous docking and MD simulation protocol (47) in order to improve the binding affinity evaluation and, thus, facilitate the selection of the best-binding peptide candidates.

It has been suggested by Fleischmann et al. (69) that high-affinity peptides close the binding groove tightly while low-affinity peptides widen the MHC-I binding groove. In our study, only two of the predicted epitope peptides were closing the groove as they reduced the F pocket size somewhat: QAEPDRAHY (from HPV type 16 E7 protein) and VYCKQQLLR (from HPV type 16 E6 protein) (Table 5). However, the predicted Prime-MMGBSA binding energies were very low for these peptides. On the other hand, none of the experimentally determined peptide-MHC-I complexes closed the groove but they also widened the groove to variable extent, PDB IDs 2BCK and 5HHP the least. Thus, the F pocket size might not be a very reliable parameter to indicate the peptide binding affinity in all cases.

The RMS fluctuations of the peptides in the crystal complexes confirm the common pattern of epitope binding to MHC-I proteins as the N- and C-terminal ends of the peptides (especially residues at P2 and P9 positions) are in general tightly bound and the residues at positions P4–P6 are generally pointing upwards to be able to interact with the T-cell receptor (70). Many of the predicted epitopes follow this binding pattern (e.g., CYSLYGTTL, EYRHYCYSL, FAFKDLFVV, AEPQRHTML). On the other hand, peptides whose C-terminus is not tightly bound are likely not good candidates for peptide vaccine development; for example the E7 viral protein peptides from HPV16 (Figure S23), and VYCKQQLLR from E6 protein of HPV16 whose positively charged arginine residue at P9 is completely out of the binding pocket (Figure S9D).

The accuracy of docking of the peptides is of crucial importance. In has been shown for the peptides binding to MHC-I HLA-A^*^24:02 that the tyrosine at P2 position of the peptide forms a hydrogen bond interaction with the His70 of the MHC protein (54). In the respective docked complexes, this interaction was not present (not even after MD) although the residues at P2 were well buried in the binding pocket. A proper docking pose with this particular hydrogen bond interaction in place might have increased the initial (and final) MMGBSA binding energies of these vaccine candidate peptides.

In various studies, immunoinformatics analyses have been performed for the prediction of antigenic epitopes against early proteins encoded by high-risk HPV genomes. Yao et al. (71) reported E6 and E7 CTL epitope prediction of HPV-16 based on distributions of HLA-A loci across populations and concluded that a combination of four peptides (FAFRDLCIVYR_52−62_ and PYAVCDKCLKF_66−76_ of E6; HGDTPTLHEY_2−11_ and YMLDLQPETT_11−20_ of E7) could vaccinate more than 50% of all individuals worldwide. The two E6 peptides are among our results as well. Subramanian and Chinnappan (72) implemented immunoinformatics, to aid in the development of a therapeutic HPV vaccine, by identifying promiscuous epitopes among E6 proteins of high risk HPVs (i.e., HPV-16, HPV-18, and HPV-45) and concluded the following fragments as the most promiscuous epitopes: FAFRDL and KLPDLCTEL. Both fragments are also found in Table 4.

There are also experimental studies that demonstrate the immunogenicity of various identified antigenic peptides of E6 and E7 proteins of HPV16/18. These include some of the peptides or peptide fragments that we have predicted in the present study, which supports the immunogenic potential of these predicted peptides. Specifically, Grabowska et al. (73) reported MHC-II 15-mer peptides of HPV16 that elicited CD4^+^ T-cell immune responses in individuals carrying the particular MHC-II alleles. These promiscuous peptides may also harbor MHC-I binding epitopes; for example E6 protein epitopes 42–56, 54–68, 74–88, and 92–106 include peptide stretches from all the studied E6 HPV16 epitopes, the longest ones being VYDFAFRD and EYRHYCY. Likewise, from the E7 protein epitopes 12–26, 64–78, and 71–85 the first 15-mer includes LQPETTDLY. Interestingly, Steinbach et al. (74) also showed the presence of HPV16 E7 protein peptides 11–19 and 11–20 on MHC-I HLA-A^*^02:01 molecules on the CaSki cell surface by mass spectrometry. These include the predicted epitope fragment LQPETT. This particular fragment was also part of the HPV16 E7 12–20 peptide that was used to treat patients with HPV16-positive neoplasia in a vaccine trial (75). On the other hand, HPV16 positive subjects have shown a positive T-cell response to the HPV16 E7 46–70 region (76). Also van der Burg et al. (77) identified this highly immunogenic region of HPV16 E7 41–62 that includes our CTL epitope QAEPDRAHY. Gallagher et al. (78) identified 15-mer peptide sequences from E6 HPV16 and 18 as candidate CD4^+^ epitopes, such as HPV16 E6 85–99 that includes HYCYSL and HPV18 E6 43–57 that includes FAFKDLFVV. Interestingly, Matijevic et al. (79) described significant CD8^+^ T-cell responses to the HPV18 E7 LFLNTLSFV peptide in HLA-A2+ clinical trial subjects receiving amolimogene (microparticle encapsulated plasmid DNA expressing antigenic regions of HPV16 and 18). That peptide includes the peptide fragment LFLNTL that is part of the predicted CTL epitope FQQLFLNTL.

Many of these reported epitopes were 15-mers or longer stretches. It has been reported that peptides with the length of 20 amino acids or longer tend to elicit immune response with less chances of inducing tolerance that results from peptide vaccination. Further, due to longer size, they may harbor more than one epitope having specificity for various MHC molecules. Longer peptides require degradation by proteolysis and only after that will they be presented by professional antigen-presenting cells such as dendritic cells; this ensures sufficient co-stimulation (24). CTL tolerization can result in enhancement of tumor outgrowth; however, presentation of peptides on dendritic cells is known to elicit immune response to peptide antigens and hence abate the effect of CTL tolerization (80). Thus, nonapeptides alone are likely not the ideal vaccine candidates but could be introduced to cells as part of longer sequence stretches or together with other longer peptides. In addition, the effectiveness and immunogenicity of peptide vaccines could be enhanced by combining them with other immunomodulatory drugs or standard cancer therapy (22).

## Conclusions

Current prophylactic HPV vaccines boost the antibody production and thus work only for those who have not been exposed previously. On the other hand, therapeutic HPV vaccines targeting E6 and E7 proteins can potentially target virus-infected cells and tumors by activating cell-mediated immunity. In this work, we combined immunoinformatics and molecular modeling approaches to predict suitable antigenic peptides for therapeutic HPV vaccine development. We identified some candidate peptides (e.g., E6 peptides FAFKDLFVV of HPV18 and CYSLYGTTL of HPV16, and E7 peptides QAEPDRAHY of HPV16 and AEPQRHTML of HPV18) that could be used in the development of therapeutic HPV vaccines. Further, we also developed our docking and MD simulation approach to improve the evaluation of the crucial epitope binding affinities by MHC-I-biased peptide docking, detailed MD simulation analysis and binding free energy calculations of the peptide-MHC-I complexes.

## Author Contributions

BJ, SR, AA, MUM, SZS, MV, MM, IJ, and MAR performed the immunoinformatics and molecular docking analyses. OMHS-A performed the molecular dynamics simulations analysis. BJ, SR, AA, and OMHS-A wrote the manuscript. OMHS-A, AA, SR, and MI critically reviewed the manuscript. All the authors approved the final manuscript.

### Conflict of Interest Statement

The authors declare that the research was conducted in the absence of any commercial or financial relationships that could be construed as a potential conflict of interest.
