# Supplementary material for: Antigenic Peptide Prediction From E6 and E7 Oncoproteins of HPV Types 16 and 18 for Therapeutic Vaccine Design Using Immunoinformatics and MD Simulation Analysis
Source: Front Immunol. 2018 Dec 19;9:3000. doi: 10.3389/fimmu.2018.03000 (PMC6305797; doi:10.3389/fimmu.2018.03000)
Supplement: Supplementary file 1 [file Data_Sheet_1.pdf]

Supplementary Material

Antigenic peptide prediction from E6 and E7 oncoproteins of HPV 16 and 18 for therapeutic vaccine design using immunoinformatics and MD simulation analysis

Basit Jabbar,[1] Shazia Rafique,[1]* Outi M. H. Salo-Ahen,[2,3]* Amjad Ali,[4] Mobeen Munir,[5] Muhammad Idrees,[1,6] Muhammad Usman Mirza,[7] Michiel Vanmeert,[7] Syed Zawar Shah,[1] Iqra Jabbar,[8] and Muhammad Adeel Rana[9]

[1]Centre of Excellence in Molecular Biology, University of the Punjab, Lahore 87-West Canal Bank Road, Thokar Niaz Baig, Lahore, Pakistan

[2]Structural Bioinformatics Laboratory, Faculty of Science and Engineering, Biochemistry, Åbo Akademi University, Turku, Finland

[3]Pharmaceutical Sciences Laboratory, Faculty of Science and Engineering, Pharmacy, Åbo Akademi University, Turku, Finland

[4]Department of Genetics, Hazara University, Mansehra, Khyber Pakhtunkhwa, Pakistan

[5]Division of Science and technology, University of Education Lahore, Pakistan

[6]Vice Chancellor, Hazara University, Mansehra, Khyber Pakhtunkhwa, Pakistan

[7]Department of Pharmaceutical and Pharmacological Sciences, Rega Institute for Medical Research, Medicinal Chemistry, University of Leuven, Leuven B-3000, Belgium

[8]School of Biological Sciences, University of the Punjab, Lahore, Pakistan

[9]Department of Microbiology Quaid-i-Azam University, Islamabad, Pakistan

*Corresponding authors:
Shazia Rafique, Centre of Excellence in Molecular Biology, University of the Punjab, 87-West Canal Bank Road, Thokar Niaz Baig, Lahore-53700, Pakistan; Tel: +92-42-5293141; Fax: +92-42-5293149; E-mail: shazia.rafique@cemb.edu.pk.

Outi M. H. Salo-Ahen, Pharmaceutical Sciences Laboratory, Faculty of Science and Engineering, Pharmacy, Åbo Akademi University, Tykistökatu 6 A, Turku, Finland; Tel. +358-2-2154009; E-mail: outi.salo-ahen@abo.fi

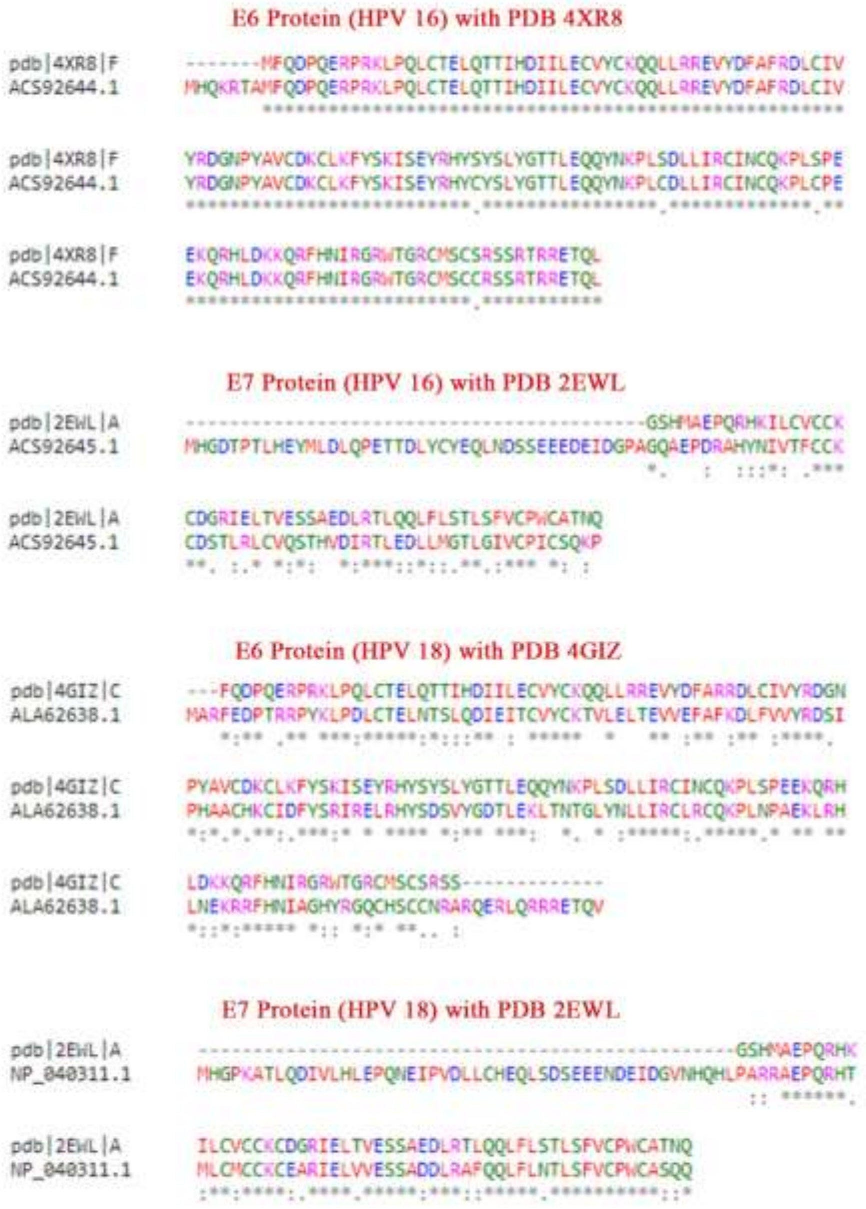

Fig. S1: Pairwise alignments of HPV early proteins with respective template sequences.

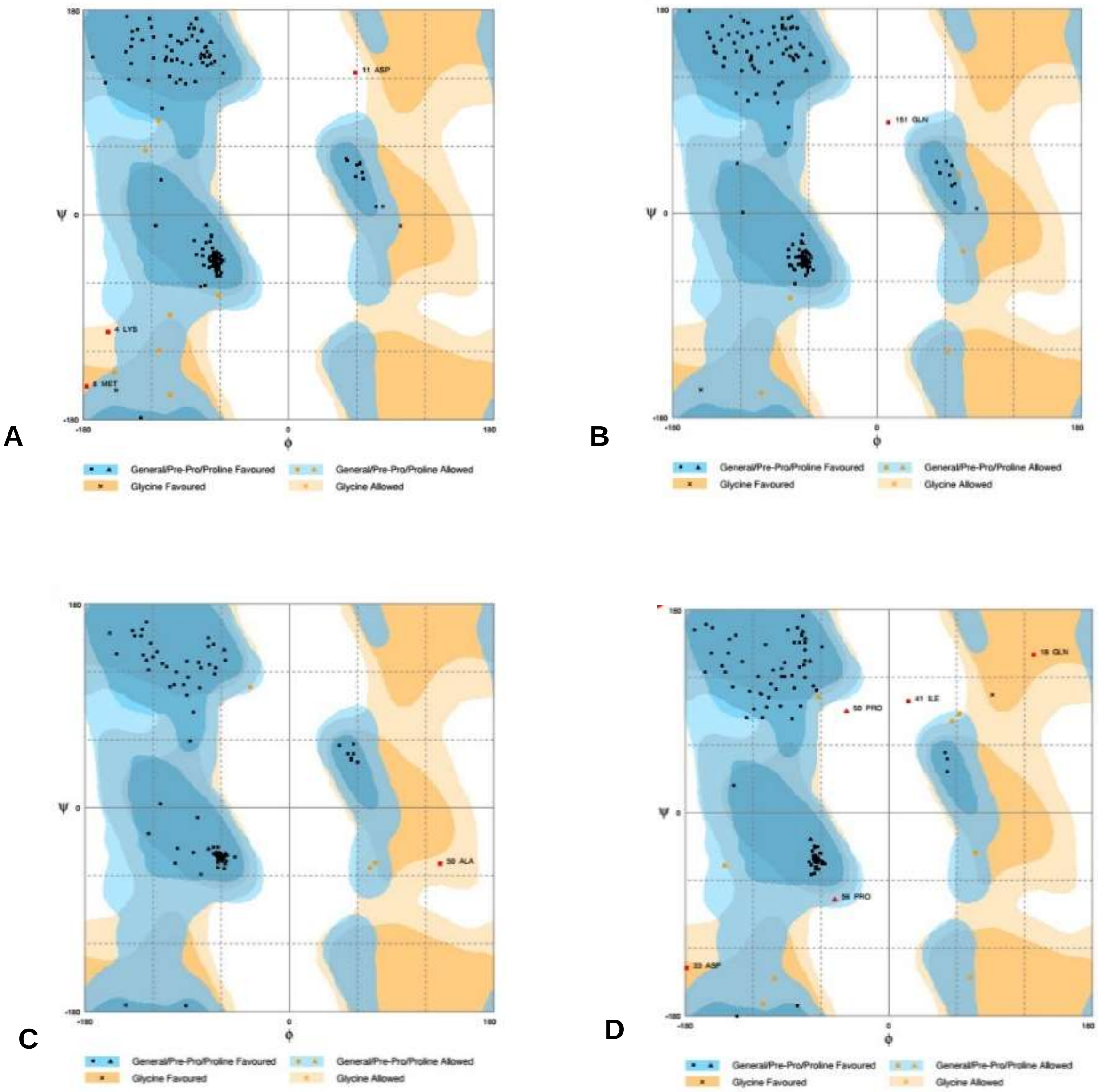

Fig. S2: Ramachandran plots indicating the stereochemical quality of the 3D structures of the HPV protein models. A) E6 protein (HPV16); B) E6 protein (HPV 18); C) E7 protein (HPV 16); D) E7 protein (HPV 18).

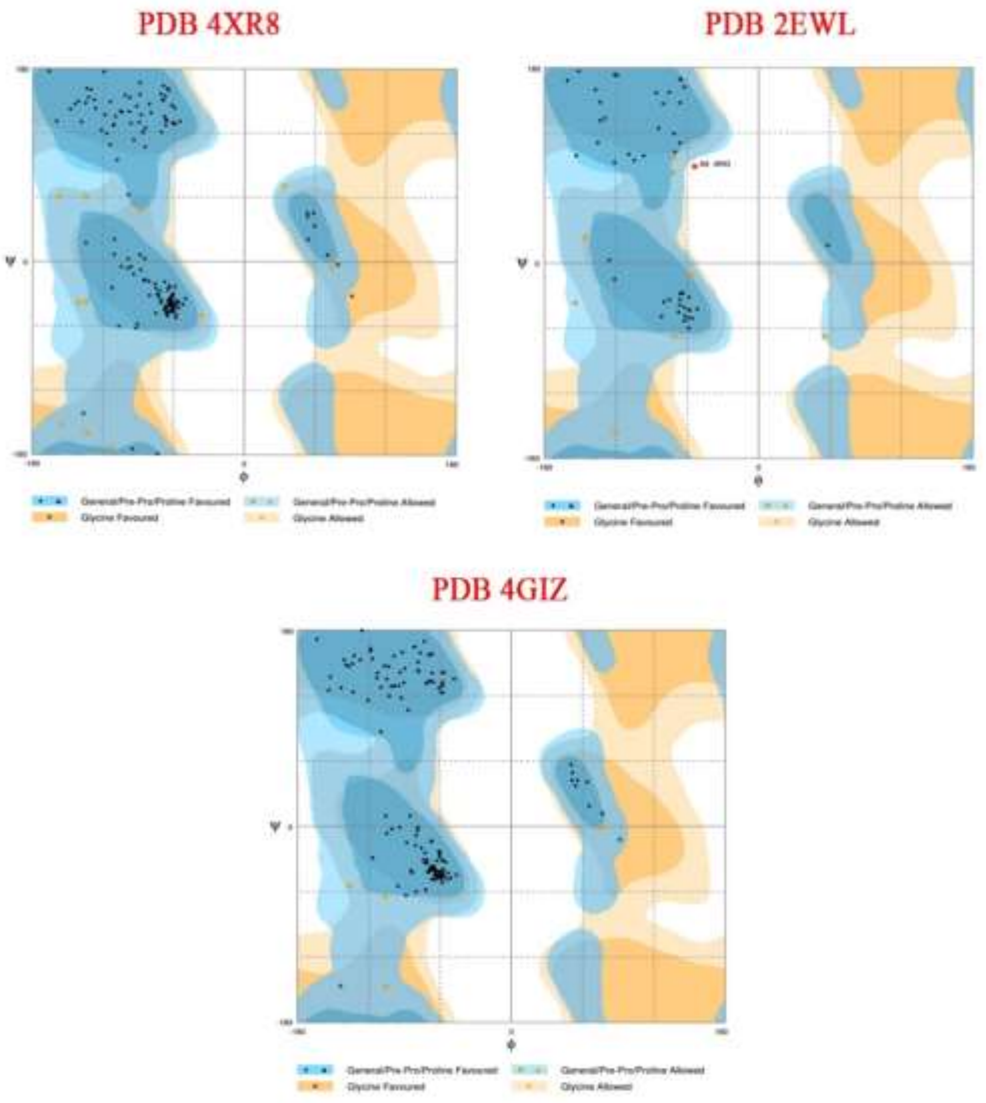

Fig. S3: Ramachandran plots of the templates that were used in the study for modeling the early proteins of HPV type 16 and 18. The crystal structure PDB ID: 4XR8, chain F had 100% residues, the NMR structure PDB ID: 2EWL had 98.9% residues while the crystal structure PDB ID: 4GIZ had 100% residues in favored and allowed regions.

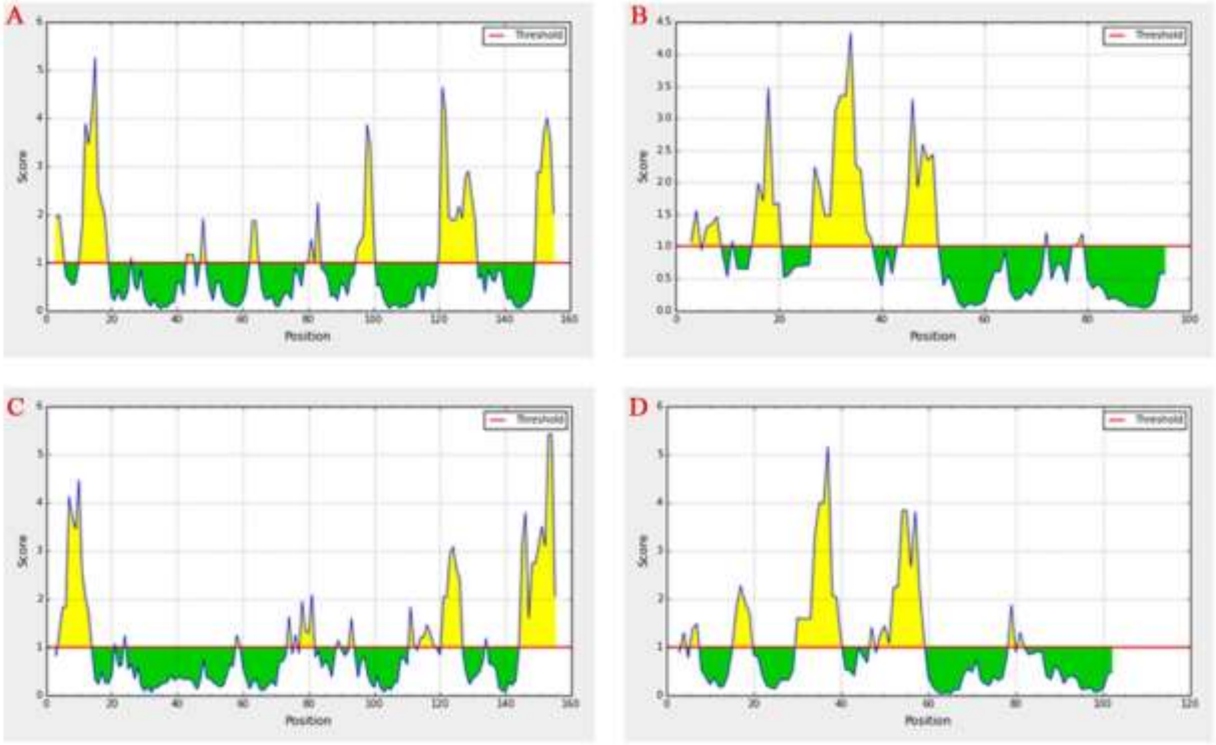

Fig. S4: Surface accessibility profile of amino acids belonging to A) E6 Protein (HPV type 16), B) E7 Protein (HPV type 16), C) E6 Protein (HPV type 18) and D) E7 Protein (HPV type 18).

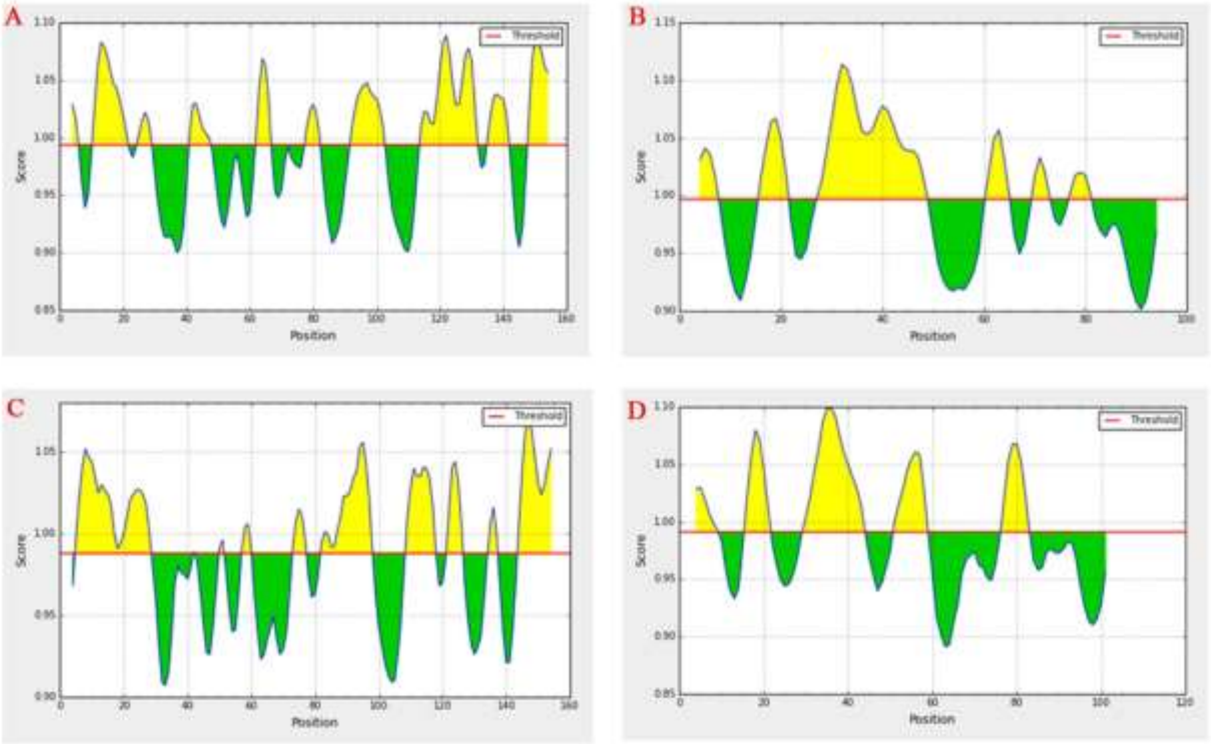

Fig. S5: Graphical representation of flexibility prediction results for A) E6 Protein (HPV type 16); B) E7 Protein (HPV type 16); C) E6 Protein (HPV type 18); and D) E7 Protein (HPV type 18).

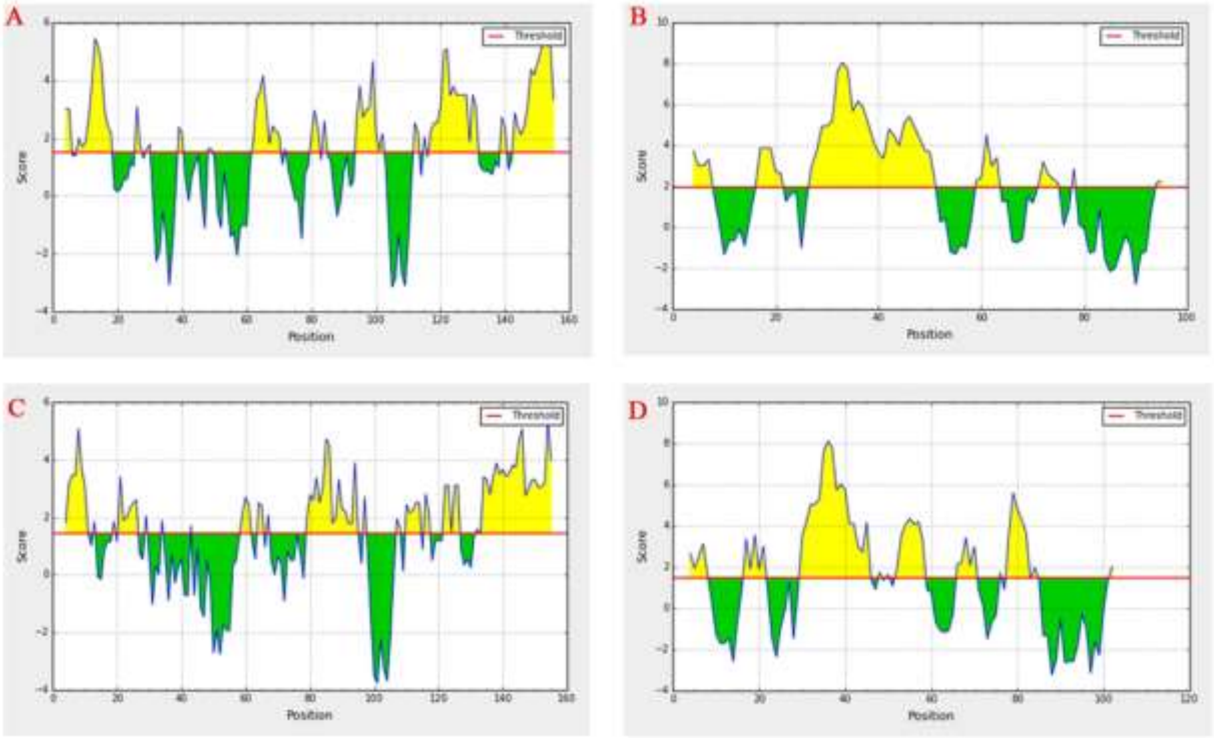

Fig. S6: Graphical representation of hydrophilicity prediction results for A) E6 Protein (HPV type 16); B) E7 Protein (HPV type 16); C) E6 Protein (HPV type 18); and D) E7 Protein (HPV type 18).

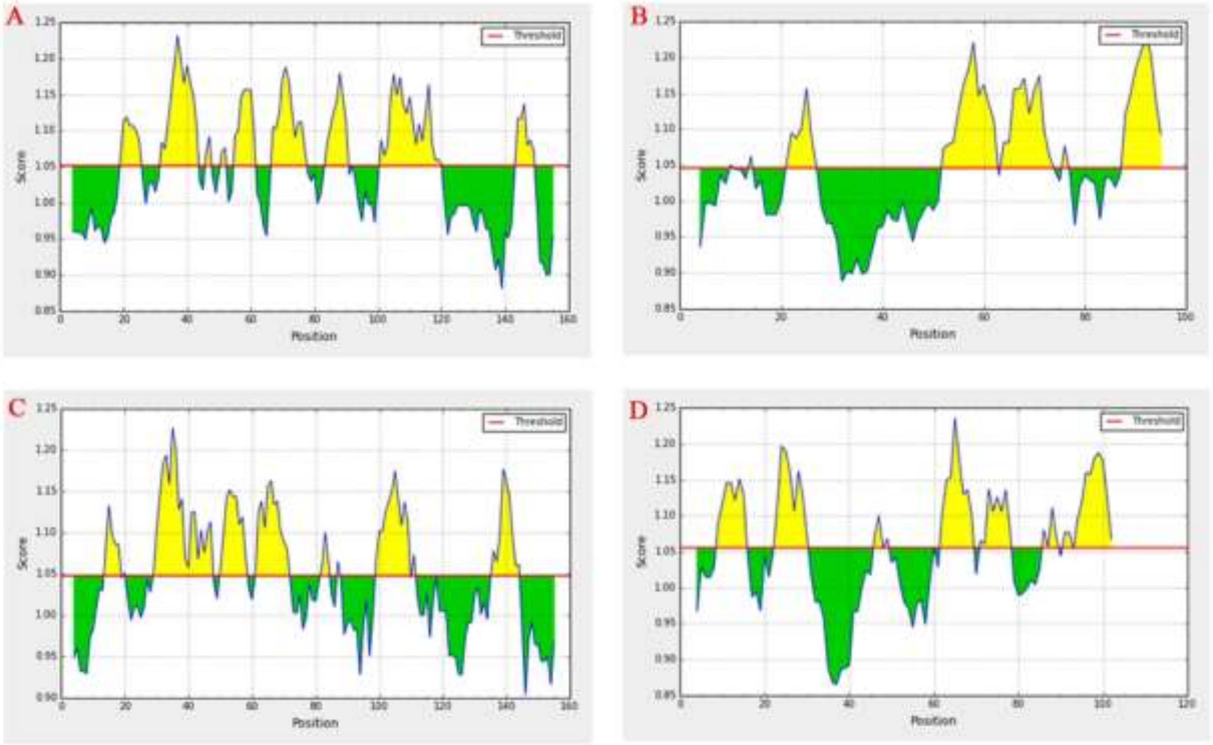

Fig. S7: Graphical Representation of predicted Linear B-Cell Epitopes of A) E6 Protein (HPV type 16), B) E7 Protein (HPV type 16), C) E6 Protein (HPV type 18) and D) E7 Protein (HPV type 18).

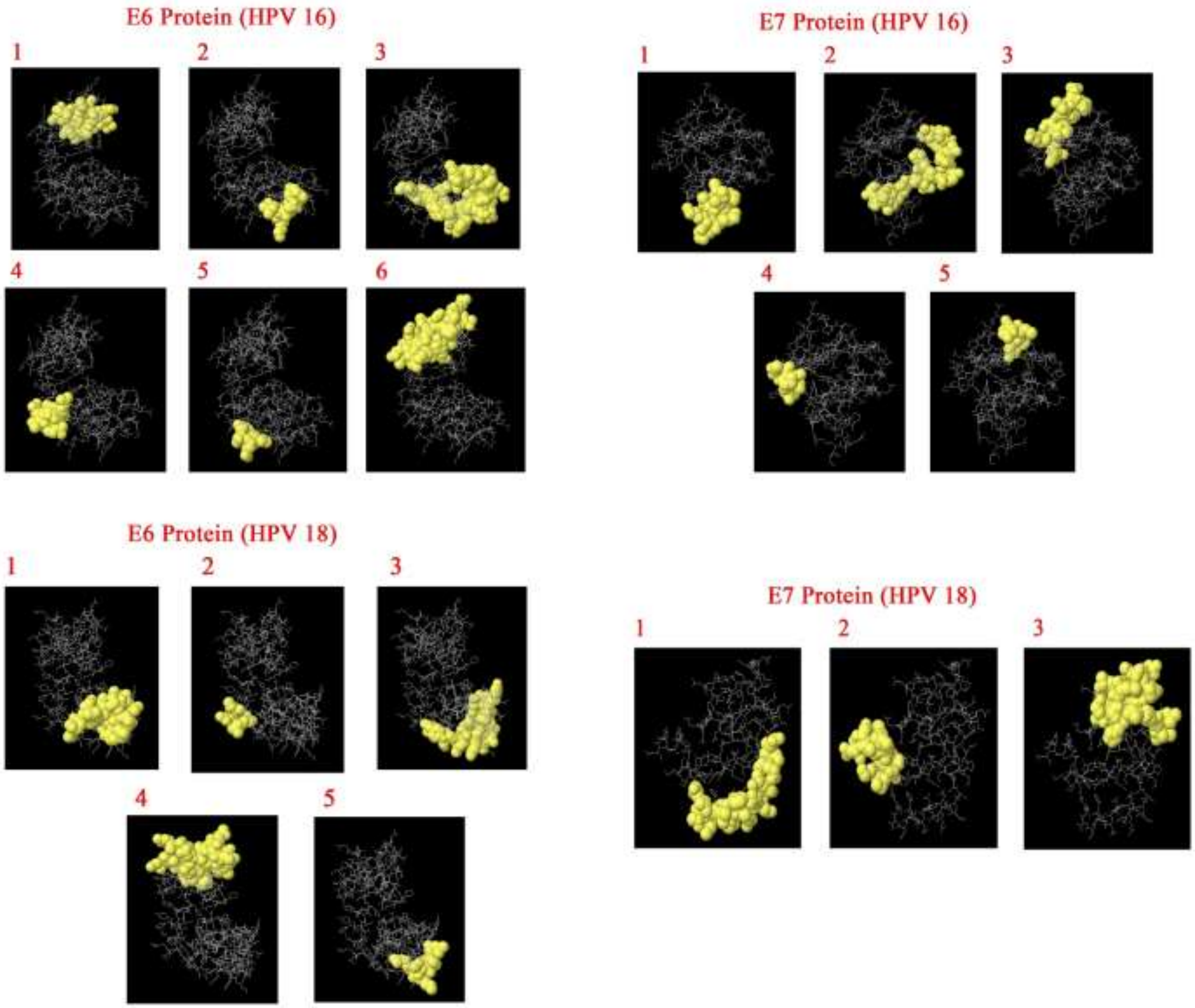

Fig. S8: ElliPro predicted conformational (discontinuous) B-cell epitopes of E6 and E7 proteins of HPV (type 16 and 18) represented in 3D. The numbers of the predicted epitope regions for each protein correspond to the number of prediction in Table 3. Orientation of the proteins is the same as in Figure 1.

Table S1: Docking results indicating output parameters of E6 and E7 protein (HPV 16/18) peptides with HLA molecules and hydrogen bonding interactions before and after MD simulations.

**Peptide Sequence**	**Pepfold3**	**FlexPepDock**			**H-bond Interactions**[a]		
	**sOPEP**	**Total score (Rosetta energy)**	**Interface energy**	**H-bond energy (sidechain-sidechain)**	**HLA-Peptide Atom Pair**	**d_{init} (Å)**	**d_{MD} (Å)**
**E6 Protein (HPV 16) peptides docked to HLA-A*24:02**							
**CYSL**YGTTL	-59.33	-405.90	-19.83	-17.63			
	Residue pairs involved in steric clashes: N/A				GLU63:OE2 - CYS1:N	2.86	2.67
					ASN77:ND2 - GLY6:O	2.73	nd
					TYR84:OH - LEU9:O	2.93	nd
					TYR116:OH - TYR5:O	2.43	nd
					THR143:OG1 - LEU9:O	2.78	nd
					TRP147:NE1 - THR8:O	2.81	2.88
					LYS146:NZ - LEU9:OXT	nd	2.77
					GLU62:OE2 - CYS1:N	nd	2.72
					GLU63:OE1 - CYS1:N	nd	2.84
					LYS66:NZ - TYR2:O	nd	2.87
					ASN77:ND2 - THR8:OG1	nd	3.02
					THR163:OG1 - SER3:OG	nd	2.84
					THR73:O - THR7:OG1	nd	2.69
					ASN77:OD1 - THR8:N	nd	3.29
					ASN77:OD1 - LEU9:N	nd	3.09
					HIS151:CD2 - TYR5:OH	nd	3.78
					TYR159 - SER3:OG	nd	3.41
VYDFAF**RDL**	-85.37	-417.00	-19.63	-19.99			
	Residue pairs involved in steric clashes: N/A				GLU63:OE1 - VAL1:N	2.71	nd
					THR143:OG1 - ASP8:O	2.84	nd
					GLU63:OE2 - TYR2:N	2.88	2.81
					ILE124:O - ARG7:NH1	3.38	nd
					HIS114:O - ARG7:NH2	3.20	nd
					GLN156:OE1 - ARG7:NH2	3.26	nd
					LYS146:NZ - ASP8:OD1	nd	2.90
					LYS146:NZ - LEU9:OXT	nd	2.84
					GLU63:OE2 - VAL1:N	nd	2.70
					LYS66:NZ - TYR2:O	nd	2.91
					THR143:OG1 - LEU9:O	nd	2.63
					GLN155:NE2 - PHE6:O	nd	2.89
					THR163:OG1 - ASP3:OD2	nd	2.80
					ASN77:OD1 - LEU9:N	nd	3.18
EY**RHYCYSL**	-75.29	-406.43	-18.86	-26.14			
	Residue pairs involved in steric clashes: N/A				GLU62:OE1 - ARG3:NH1	2.87	nd
					TYR171:OH - GLU1:OE1	2.94	nd
					TYR159:OH - HIS4:ND1	2.89	nd
					HIS70:NE2 - TYR5:N	3.23	nd
					HIS70:O - TYR7:OH	2.79	nd
					ASP74:OD1 - TYR7:OH	2.84	nd
					TYR171:OH - GLU1:N	4.07	nd
					HIS114 - TYR5:OH	3.08	nd
					LYS66:NZ - GLU1:OE2	nd	2.82

					LYS146:NZ - LEU9:O	nd	2.87
					ARG170:NH1 - GLU1:OE1	nd	2.89
					ARG170:NH1 - GLU1:OE2	nd	3.11
					GLU63:OE1 - GLU1:N	nd	2.78
					TRP147:NE1 - SER8:O	nd	2.91
					THR163:OG1 - :HIS4:O	nd	2.79
					HIS114:ND1 - TYR5:OH	nd	3.36
					GLN156:OE1 - TYR5:OH	nd	2.79
					ASP74:OD2 - TYR7:OH	nd	2.67
					ASN77:OD1 - LEU9:N	nd	3.16
DFAF**RDLCI**	-73.82	-414.94	-22.64	-19.08			
	Residue pairs involved in steric clashes: N/A				GLU62:OE1 - ASP1:N	3.44	nd
					GLU63:OE2 - ASP1:N	2.87	2.86
					TRP147:NE1 - ASP6:O	3.04	nd
					GLU63:OE2 - PHE2:N	2.92	nd
					HIS70:NE2 - PHE4:N	2.99	nd
					ASN77:ND2 – ILE9:OXT	3.10[b]	nd
					LYS146:NZ - ILE9:O	nd	2.74
					ARG170:NH1 - ASP1:OD2	nd	2.80
					ARG170:NH2 - ASP1:OD2	nd	2.92
					GLU63:OE1 - ASP1:N	nd	2.75
					ASP74:OD2 - ARG5:NH2	nd	2.78
					TYR59:OH - ASP1 :OD1	nd	2.71
					HIS70:NE2 - ARG5:O	nd	3.39
					TYR116:OH- LEU7:O	nd	2.72
					TRP147:NE1 - CYS8:O	nd	2.83
					GLN156:NE2 - ASP 6:OD1	nd	2.84
					TYR159:OH - ARG5:O	nd	3.07
					ARG170:NH2 - ASP1:O	nd	3.37
					TYR171:OH - ASP1:OD2	nd	2.68
					GLU63:OE1 - PHE2:N	nd	3.40
					TYR159:OH - PHE4:N	nd	2.97
					TYR159:OH - ARG5:N	nd	3.06
					TYR116:OH - ARG5:NE	nd	3.13
					THR73:OG1 - ARG5:NH1	nd	3.08
					HIS70:O - ARG5:NH2	nd	3.04
					THR73:OG1 - ARG5:NH2	nd	2.91
					ASN77:OD1 - ILE9:N	nd	2.99
P**YAVCDKCL**	-63.04	-433.54	-19.97	-21.21			
	Residue pairs involved in steric clashes: N/A				GLU63:OE2 - PRO1:N	2.80	nd
					SER9:OG - TYR2:OH	2.90	nd
					TRP147:NE1 - CYS8:O	2.60	3.08
					GLN155:NE2 - ASP6:OD2	2.90	nd
					HIS114:NE2 - LYS7:NZ	3.29	nd
					LYS146:NZ - LEU9:OXT	nd	2.88
					GLU63:OE1 - PRO1:N	nd	2.74
					HIS70:NE2 - CYS5:O	nd	3.05
					THR73:OG1 - ASP6:OD1	nd	2.68
					THR143:OG1 - LEU9:OXT	nd	2.64
					ALA24:O - TYR2:OH	nd	3.21
					GLN156:OE1 - CYS5:N	nd	3.07
**VYCKQQ**LLR	-67.65	-413.84	-19.23	-20.93			

	Residue pairs involved in steric clashes: N/A				LYS146:NZ - ARG9:O	3.95	2.91
					GLU76:OE2 - ARG9:NH2	2.89	nd
					GLN156:NE2 - LYS4:O	2.79	2.85
					GLU62:OE1 - TYR2:OH	2.81	nd
					HIS70:NE2 - GLN5:N	3.01	nd
					GLU76:OE2 - ARG9:NE	2.89	nd
					HIS70 - GLN5:N	3.66	nd
					HIS70 - GLN5:NE2	3.90	3.22
					LYS66:NZ - CYS3:O	nd	2.95
					ASN77:ND2 - LEU8:O	nd	2.89
					TRP147:NE1 - LEU8:O	nd	2.99
**E7 Protein (HPV 16) peptides docked to HLA-A*01:01**							
QAEPDRAH**Y**	-46.20	-383.58	-16.838	-20.23			
	Residue pairs involved in steric clashes: N/A				ARG114:NH1 - TYR9:OXT	2.76	nd
					ARG114:NH2 - TYR9:OXT	2.76	nd
					ARG163:NH2 - GLU3:OE2	3.75	nd
					GLU63:OE2 - GLN1:N	2.79	2.72
					ASN66:ND2 - GLU3:OE2	2.98	nd
					ASN77:ND2 - TYR9:O	3.38	nd
					ARG163:NE - GLN1:OE1	2.91	nd
					ARG163:NH2 - GLN1:OE1	2.80	nd
					TYR171:OH - GLN1:N	3.40	nd
					GLN62:OE1 - GLN1:NE2	2.75	nd
					ALA152:O - ARG6:NH2	2.85	nd
					GLU63:OE1 - GLN1:N	nd	2.86
					TYR59:OH - GLN1:OE1	nd	2.71
					TYR99:OH - ALA2:O	nd	2.79
					GLN155:NE2 - ASP5:OD1	nd	3.02
					TYR159:OH - GLN1:O	nd	3.12
					TYR159:OH - GLU3:O	nd	2.75
					GLU63:OE1 - ALA2:N	nd	2.81
TT**DLYCYEQ**	-57.04	-413.45	-17.29	-23.88			
	Residue pairs involved in steric clashes: N/A				ARG114:NH2 - GLU8:OE2	2.84	nd
					ASN66:ND2 - ASP3:O	2.83	nd
					ASN77:ND2 - GLU8:OE1	2.79	nd
					TYR99:OH - LEU4:O	2.96	nd
					ARG114:NH1 - CYS6:O	2.92	nd
					TRP147:NE1 - GLN9:OXT	3.22	2.86
					ARG156:NH2 - LEU4:O	2.88	3.04
					GLU63:OE2 - TYR5:OH	2.90	nd
					ASN66:O - TYR7:OH	3.29	nd
					ARG114:NH1 - GLU8:OE2	nd	2.89
					ARG114:NH2 - GLU8:OE1	nd	2.79
					LYS146:NZ - GLN9:O	nd	2.85
					GLU63:OE1 - THR1:N	nd	2.70
					ARG156:NH1 - LEU4:O	nd	2.79
					ARG156:NH2 - ASP3:O	nd	2.92
					ARG163:NE - THR1:O	nd	3.04
					ARG163:NH2 - THR1:O	nd	2.90
					TYR171:OH - THR2:OG1	nd	3.00
					ASN66:OD1 - TYR5:OH	nd	2.75
LQPETT**DLY**	-53.60	-402.81	-16.47	-23.68			

	Residue pairs involved in steric clashes: N/A				TYR171:OH - LEU1:O	2.74	nd
					GLU63:OE1 - LEU1:N	2.82	2.79
					TYR99:OH - GLN2:O	2.58	nd
					MET5:O - GLN2:NE2	3.30	nd
					ARG114:NH1 - GLU4:OE1	2.87	nd
					ARG114:NH2 - THR5:O[b]	3.52	nd
					TRP147:NE1 - ASP7:OD1	2.78	nd
					ASN77:ND2 – LEU8:O[b]	3.28	nd
					LYS146:NZ - TYR9:O	nd	2.94
					GLU55:OE1 - LEU1:N	nd	2.69
					GLU63:OE2 - LEU1:N	nd	2.76
					ASN66:ND2 - GLU4:O	nd	2.89
					ARG114:NH1 - GLU4:OE2	nd	2.72
					ARG156:NH1 - PRO3:O	nd	2.92
					ARG156:NH2 - GLN2:OE1	nd	2.73
					ARG163:NH2 - LEU1:O	nd	3.33
					GLU63:OE2 - GLN2:N	nd	3.02
					ASN66:OD1 - GLU4:N	nd	2.99
**E6 Protein (HPV 18) peptides docked to HLA-A*02:01**							
K**LPDLC**TEL	-62.38	-392.03	-22.03	-14.59			
	Residue pairs involved in steric clashes: N/A				GLU63:OE2 - LYS1:N	2.82	2.70
					GLU55:OE1 - LYS1:NZ	2.89	2.78
					LYS66:NZ - LEU2:O	2.87	3.39
					GLU63:OE1 - LEU2:N	2.99	nd
					LYS66:NZ - ASP4:OD2	nd	2.81
					LYS66:NZ - PRO3:O	nd	3.06
					ARG97:NH1 - THR7:O	nd	2.84
					ARG97:NH2 - THR7:O	nd	2.83
					THR163:OG1 - ASP4:OD1	nd	2.77
					TYR171:OH - LYS1:O	nd	2.76
					ILE52:O - LYS1:NZ	nd	2.80
					GLN54:OE1 - LYS1:NZ	nd	2.72
					GLU63:OE2 - LEU2:N	nd	2.89
FAF**KDLFVV**	-91.53	-386.78	-29.44	-15.57			
	Residue pairs involved in steric clashes: N/A				GLU63:OE2 - PHE1:N	2.88	2.66
					TYR59:OH - PHE1:N	2.67	nd
					TYR171:OH – PHE1:N[b]	2.86	nd
					TYR99:OH - ALA2:O	2.84	nd
					LYS66:NZ - LYS4:O	2.90	nd
					TYR159:OH - ASP5:OD2	3.28	nd
					ARG97:NH2 - PHE7:O	2.74	3.01
					TRP147:NE1 - VAL8:O	2.82	2.97
					THR80:OG1 - VAL9:OXT	2.86	nd
					ASP77:OD1 - VAL9:N	2.88	2.96
					TYR99:OH - PHE1:O	nd	2.72
					GLU63:OE2 - ALA2:N	nd	2.85
					TYR159:OH - ASP5:O	nd	3.48
					ARG97:NH1 - PHE7:O	nd	2.96
					LYS146:NZ – VAL9:OXT	nd	2.94
**TVLELTEVV**	-58.31	-368.79	-18.23	-15.82			
	Residue pairs involved in steric clashes: N/A				TRP147:NE1 - VAL8:O	2.87	nd
					TYR7:OH - THR1:N	3.32	nd

					HIS70:NE2 - LEU5:N	2.93	nd
					LYS66:NZ - GLU4:OE2	nd	2.88
					ARG97:NH1 - GLU7:OE1	nd	2.78
					GLU63:OE1 - THR1:N	nd	2.80
					TYR99:OH - LEU3:O	nd	2.73
					TYR59:OH - THR1:N	nd	3.26
					GLU63:OE2 - VAL2:N	nd	2.70
					TYR159:OH - GLU4:N	nd	2.98
LQDI**EITCV**	-50.24	-396.37	-17.51	-16.01			
	Residue pairs involved in steric clashes: N/A				LYS66:NZ - ASP3:OD1	2.80	nd
					HIS70:NE2 - ILE4:O	2.87	nd
					TYR84:OH - VAL9:OXT	2.85	nd
					GLN155:NE2 - GLU5:O	3.15	nd
					TYR171:OH - LEU1:O	3.35	2.78
					TYR59:OH - LEU1:N	2.63	nd
					LYS146:NZ - VAL9:O	nd	2.90
					GLU63:OE1 - LEU1:N	nd	2.74
					GLU63:OE2 - LEU1:N	nd	2.93
					TYR99:OH - GLN2:OE1	nd	2.71
					TRP147:NE1 - CYS8:O	nd	2.88
					TYR159:OH - ASP3:O	nd	2.88
					GLU63:OE1 - GLN2:N	nd	2.93
					TYR171:OH - GLN2:NE2	nd	3.06
					ASP77:OD2 - VAL9:N	nd	2.85
**KTVLELTEV**	-57.92	-397.64	-25.97	-14.61			
	Residue pairs involved in steric clashes:				LYS66:NZ - GLU5:OE1	2.84	nd
					ARG97:NH1 - THR7:O	2.87	nd
					ARG97:NH2 - THR7:O	2.80	nd
					TRP147:NE1 - GLU8:O	2.81	nd
					TYR159:OH - GLU5:OE2	2.71	nd
					TRP167:NE1 - THR2:O	2.69	nd
					HIS70:NE2 - LEU6:N	3.34	nd
					LYS66:NZ - GLU5:OE2	nd	2.81
					LYS146:NZ - VAL9:O	nd	2.82
					GLU63:OE2 - LYS1:N	3.14	3.49
					HIS70:NE2 - THR2:OG1	nd	2.95
					TRP167:NE1 - LYS1:O	nd	2.84
					ASP77:OD1 - VAL9:N	2.80	3.02
**E7 Protein (HPV 18) peptides docked to HLA-B*44:02**							
AEPQRHTM**L**	-51.56	-399.84	-19.19	-27.15			
	Residue pairs involved in steric clashes: N/A				TYR171:OH - ALA1:N	3.08	nd
					TYR7:OH - ALA1:N	3.01	nd
					TYR7:OH - ALA1:O	2.86	nd
					ARG62:NH2 - GLU2:OE1	2.84	3.32
					ARG62:NE - GLU2:OE1	2.98	nd
					TYR99:OH - GLU2:O	2.87	nd
					TYR9:OH - PRO3:O	2.58	nd
					ASP114:OD1 - ARG5:NH1	3.02	2.82
					TYR74:OH - HIS6:NE2	2.63	nd
					TRP147:NE1 - MET8:O	2.91	2.81
					LYS146:NZ - LEU9:O	2.87	nd
					ASN77:ND2 - LEU9:OXT	3.46[b]	nd

					ASP114:OD1 - ARG5:NH2	nd	2.92
					ASP156:OD2 - ARG5:NH2	nd	2.81
					ARG97:NH1 - HIS6:ND1	nd	3.23
					ARG97:NH1 - THR7:O	nd	2.87
					ARG97:NH2 - THR7:O	nd	2.85
					TYR99:OH - PRO3:O	nd	2.84
					ARG170:NH 1 - ALA1:O	nd	2.81
					ASP156:OD2 - ARG5:NE	nd	2.88
					ASN77:OD1 - LEU9:N	nd	2.98
**L**EPQNEIP**V**	-49.83	-428.33	-23.09	-23.68			
	Residue pairs involved in steric clashes: N/A				ARG97:NH2 - GLU6:OE1	-2.77	nd
					ARG62:NH1 - GLU2:O	2.67	nd
					ASN77:ND2 - PRO8:O	2.83	nd
					ASN77:ND2 - VAL9:OXT	2.84	nd
					ARG97:NE - GLU6:OE1	2.92	3.06
					ARG97:NH1 - ILE7:O	3.14	nd
					THR143:OG1 - VAL9:O	2.78	nd
					GLU63:OE2 - GLU2:N	2.89	nd
					TYR159:OH - ASN5:ND2	3.12	nd
					ASP116:OD2 - VAL9:N	3.19	nd
					LYS45:NZ - GLU2:OE1	nd	2.91
					LYS45:NZ - GLU2:OE2	nd	2.90
					LYS146:NZ - VAL9:O	nd	2.81
					GLU63:OE2 - LEU1:N	nd	2.70
					TYR74:OH - GLU6:OE1	nd	2.75
					TYR123:OH - PRO8:O	nd	2.79
					ILE66:O - GLN4:NE2	nd	3.26
**CE**A**RIELVV**	-61.40	-422.99	-13.61	-18.32			
					TRP147:NE1 - VAL8:O	2.92	nd
					TYR171:OH - CYS1:N	3.00	nd
					TYR159:OH - ARG4:NE	3.28	nd
					TYR159:OH - ARG4:NH2	3.46	nd
					LYS146:NZ - VAL9:O	nd	2.82
					GLU63:OE1 - CYS1:N	nd	2.67
					ASP156:OD1 - ARG4:NH1	nd	3.13
					ASN77:ND2 - VAL8:O	nd	3.20
					ARG97:NH2 - GLU6:O	nd	2.81
**FQQL**FLNT**L**	-71.43	-393.80	-12.97	-19.46			
	Residue pairs involved in steric clashes: N/A				GLU63:OE2 - PHE1:N	3.86	nd
					ASN77:ND2 - LEU9:OXT	2.59	nd
					ARG97:NH1 - GLN3:OE1	2.65	nd
					LYS146:NZ - THR8:O	2.93	nd
					TRP147:NE1 - THR8:O	2.95	2.88
					TRP147:NE1 - THR8:OG1	2.85	nd
					ASP114:OD2 - PHE5:N	3.08	nd
					ASN77:ND2 - PHE5:O	nd	3.36
NEIP**VDLLC**	-56.81	-430.42	-22.69	-23.89			
	Residue pairs involved in steric clashes: N/A				LYS45:NZ - GLU2:OE2	2.87	2.73
					GLU63:OE2 - ASN1:N	2.93	2.89
					TYR9:OH - GLU2:OE1	2.80	2.64
					TYR74:OH - VAL5:O	2.76	nd
					ASN77:ND2 - ASP6:OD2	2.90	3.00

		ARG97:NH2 - VAL5:O	2.84	3.08
		TYR171:OH - ASN1:N	2.95	nd
		SER167:OG - ASN1:ND2	2.89	nd
		ASN77:OD1 - LEU7:N	3.35	3.07
		ASN77:OD1 - LEU8:N	3.00	2.85
		LYS45:NZ - GLU2:OE1	nd	2.98
		GLU63:OE1 - ASN1:N	nd	2.72
		ARG97:NE - VAL5:O	nd	3.02
		LYS146:NZ - LEU7:O	nd	2.94
		LYS146:NZ - LEU8:O	nd	2.96
		TRP147:NE1 - LEU7:O	nd	3.38

[a] Hydrogen bonding interactions were measured with BIOVIA Discovery Studio (version 4.5; Accelrys Inc.). [b]H-bond or other polar interaction measured by PyMOL v. 2.1.0 (Schrödinger, LLC)

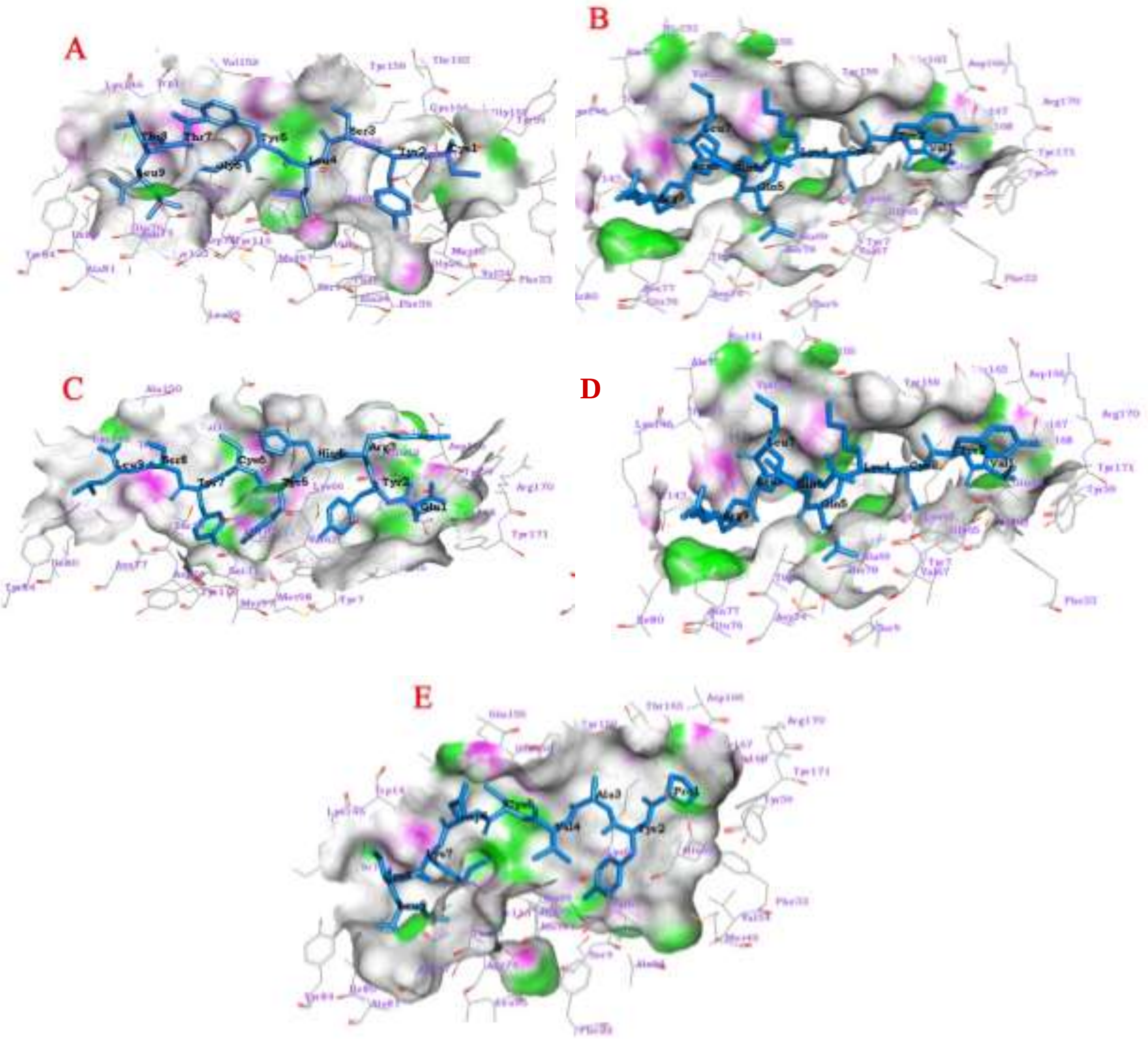

Fig. S9: E6 (HPV type 16) peptides docked at the binding pocket in the receptor HLA-A*24:02. A) CSYLYGTTL, B) VYDFAFRDL, C) EYRHYCYSL, D) VYCKQQLLR, E) PYAVCDKCL.

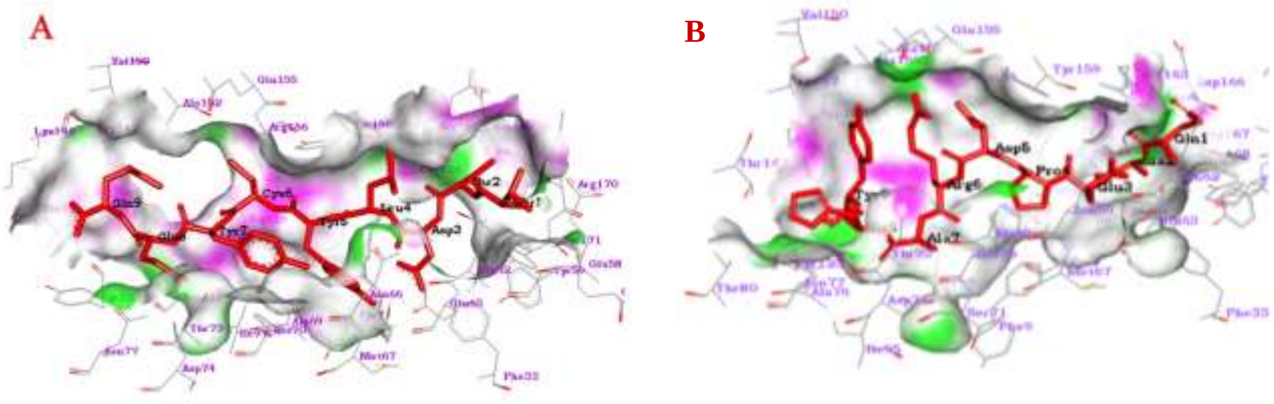

Fig. S10: E7 (HPV type 16) peptides docked at the binding pocket in the receptor HLA-A*01:01. A) TTDLYCYEQ and B) QAEPDRAHY.

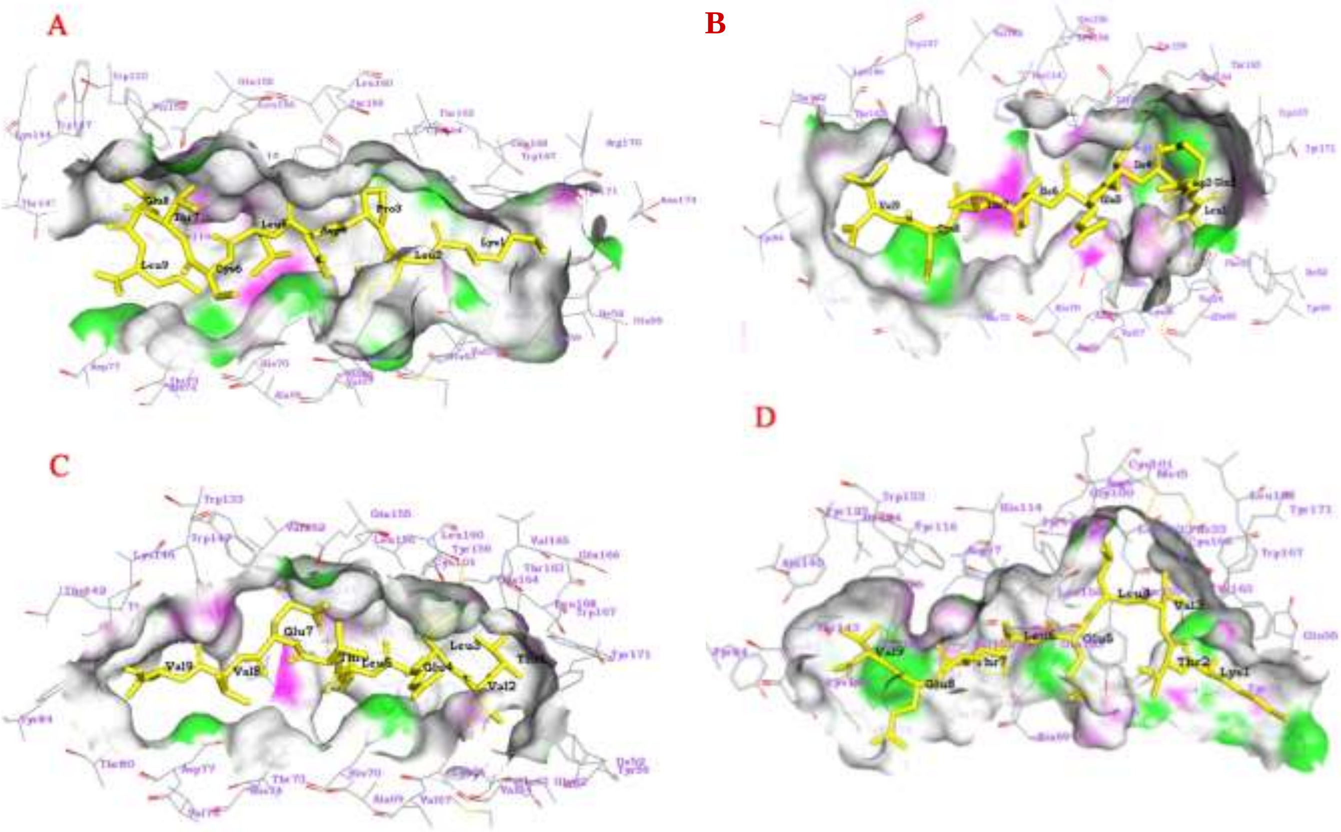

Fig. S11: E6 (HPV type 18) peptides docked at the binding pocket in the receptor HLA-A*02:01. A) KLPDLCTEL, B) LQDIEITCV, C) TVLELTEVV and D) KTVLELTEV.

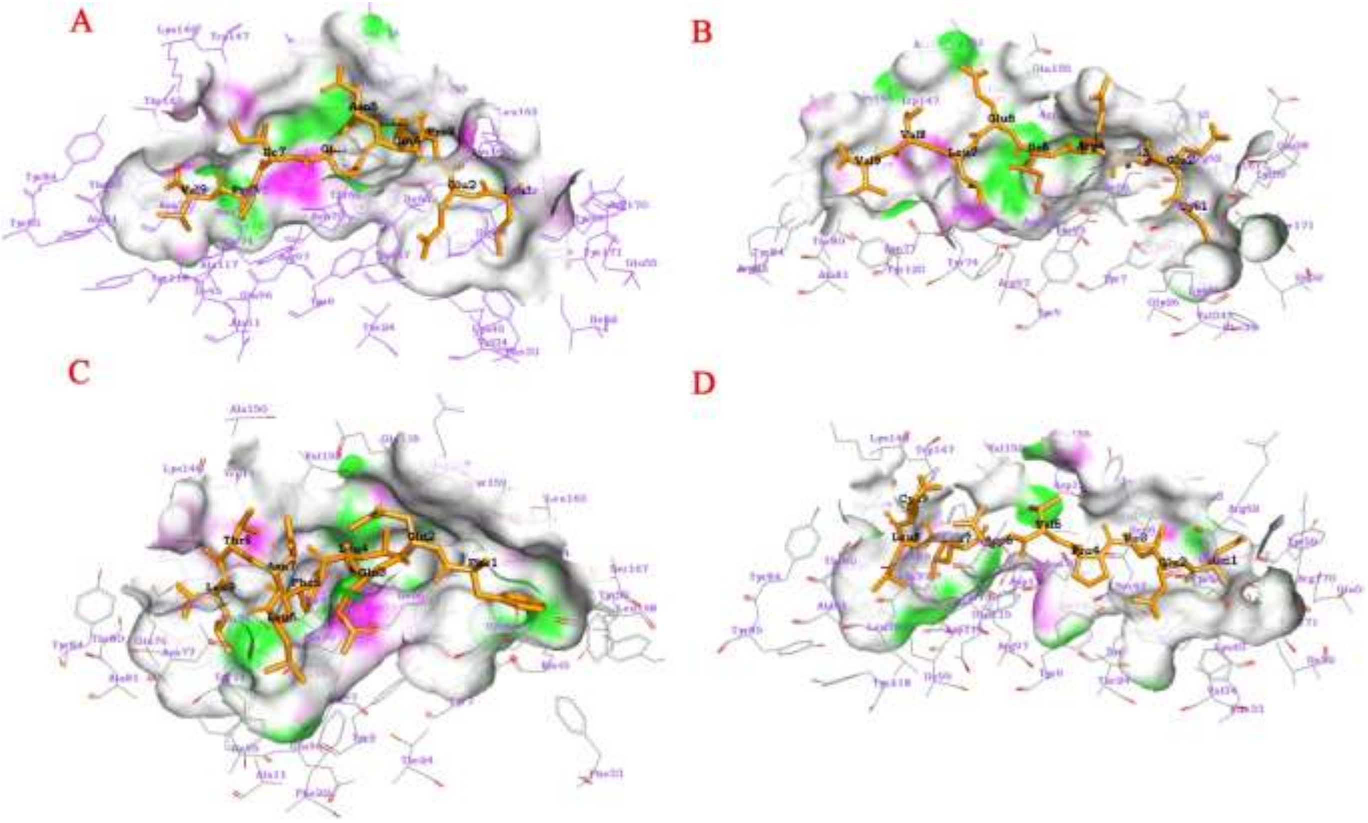

Fig. S12: E7 (HPV type 18) peptides docked at the binding pocket in the receptor HLA-B*44:02. A) LEPQNEIPV, B) CEARIELVV, C) FQQLFLNTL and D) NEIPVDLLC.

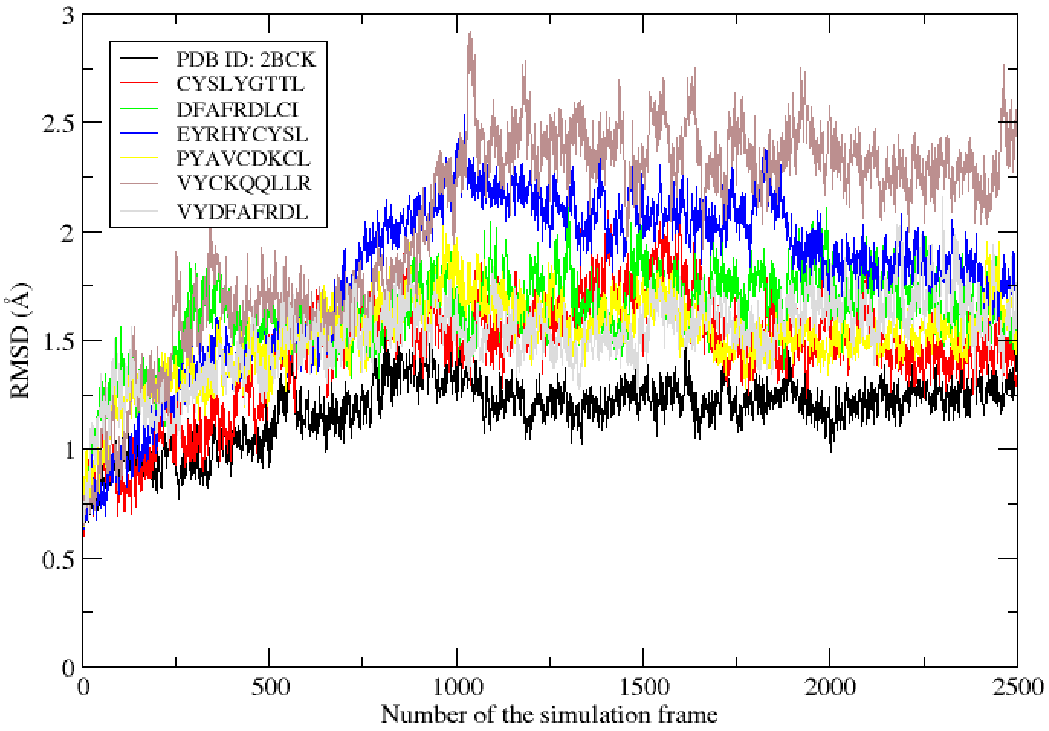

Fig. S13: RMSD of the backbone atoms of the MHC-I binding groove domain (residues 1-180 in PDB 2BCK) during the 10 ns MD simulation of the HLA-A*24:02–peptide complexes.

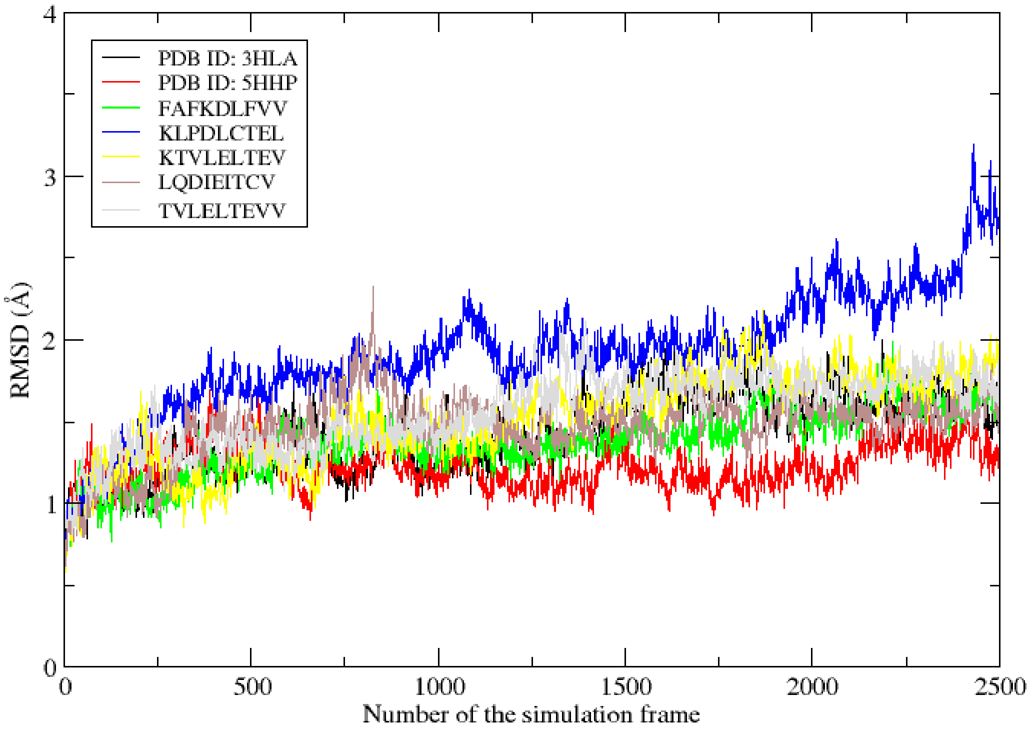

Fig. S14: RMSD of the backbone atoms of the MHC-I binding groove domain (residues 1-180) during the 10 ns MD simulation of the HLA-A*02:01–peptide complexes.

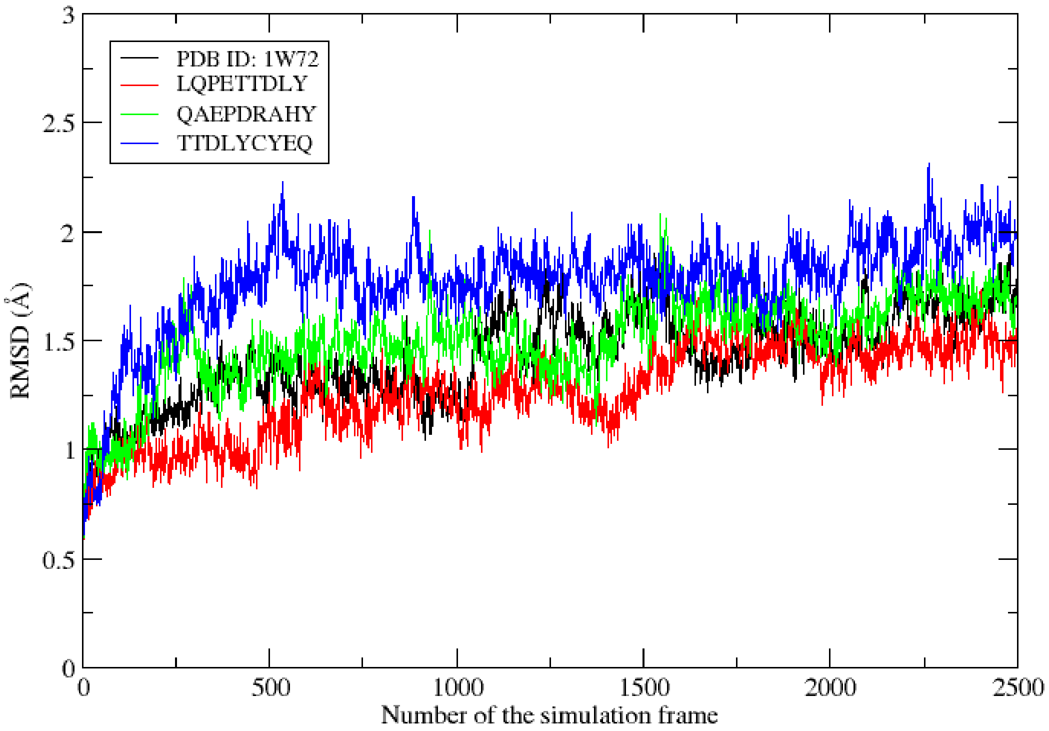

Fig. S15: RMSD of the backbone atoms of the MHC-I binding groove domain (residues 1-180) during the 10 ns MD simulation of the HLA-A*01:01–peptide complexes.

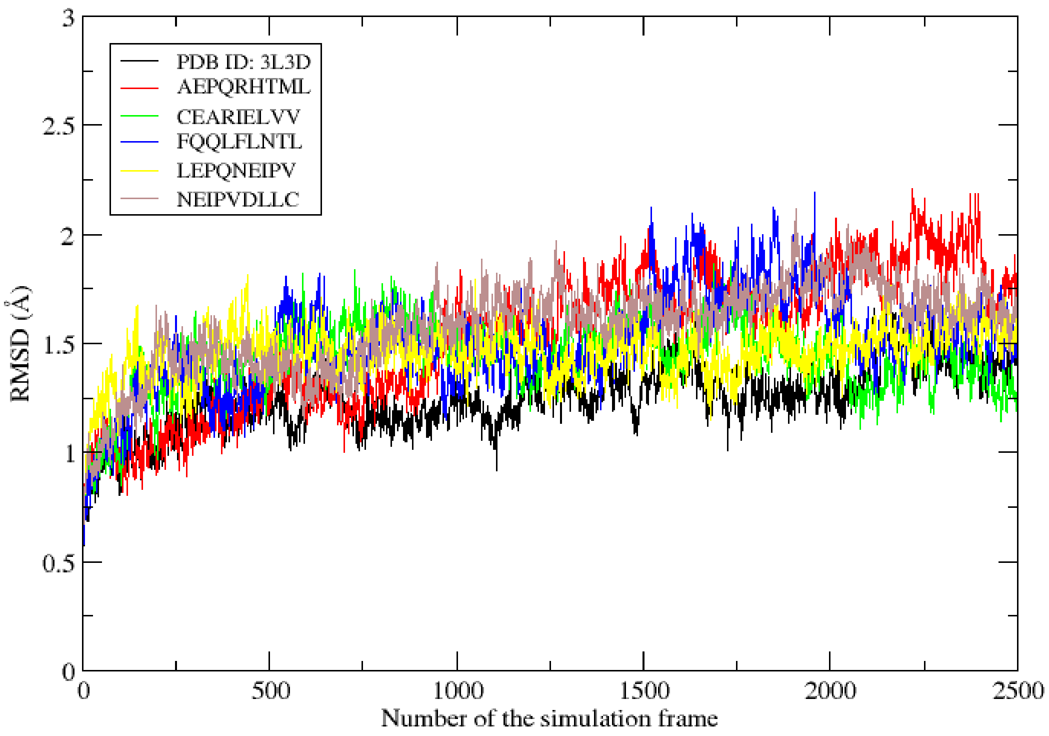

Fig. S16: RMSD of the backbone atoms of the MHC-I binding groove domain (residues 1-180) during the 10 ns MD simulation of the HLA-B*44:02–peptide complexes.

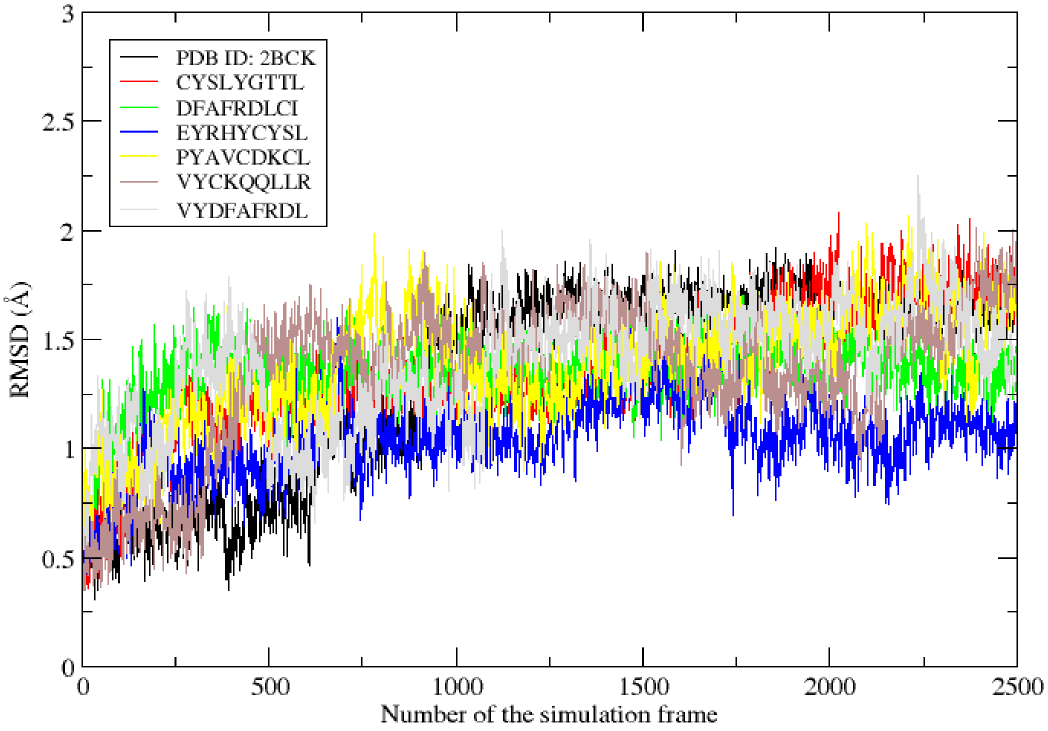

Fig. S17: RMSD of the backbone atoms of the peptide bound to HLA-A*24:02during the 10 ns MD simulation.

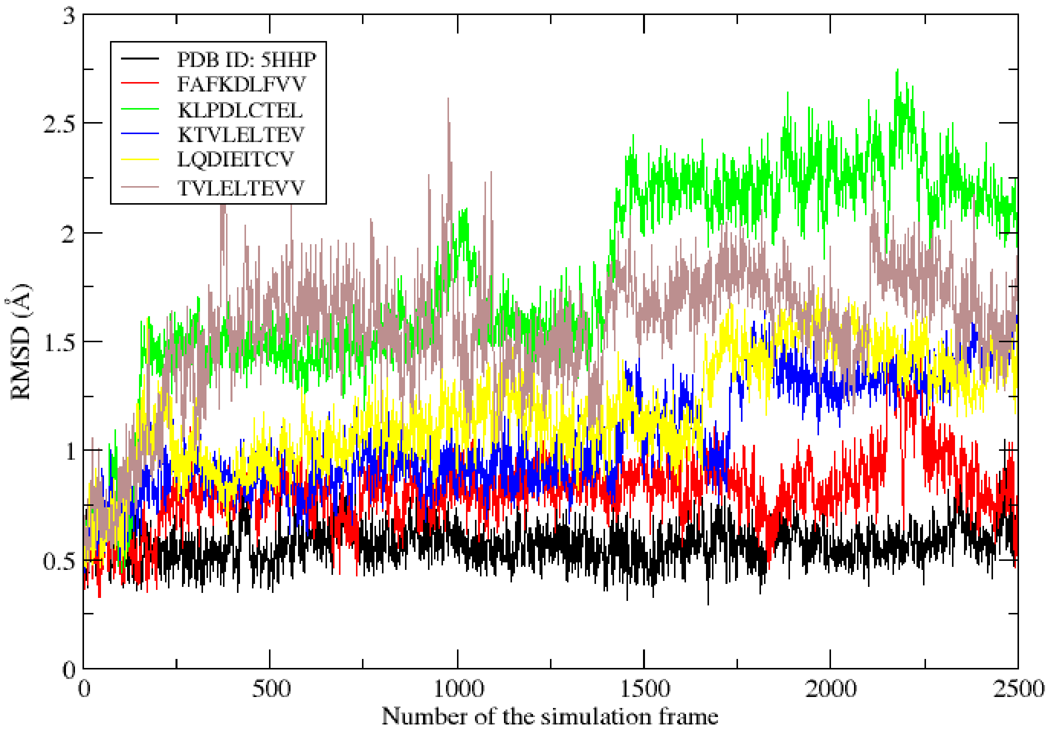

Fig. S18: RMSD of the backbone atoms of the peptide bound to HLA-A*02:01during the 10 ns MD simulation.

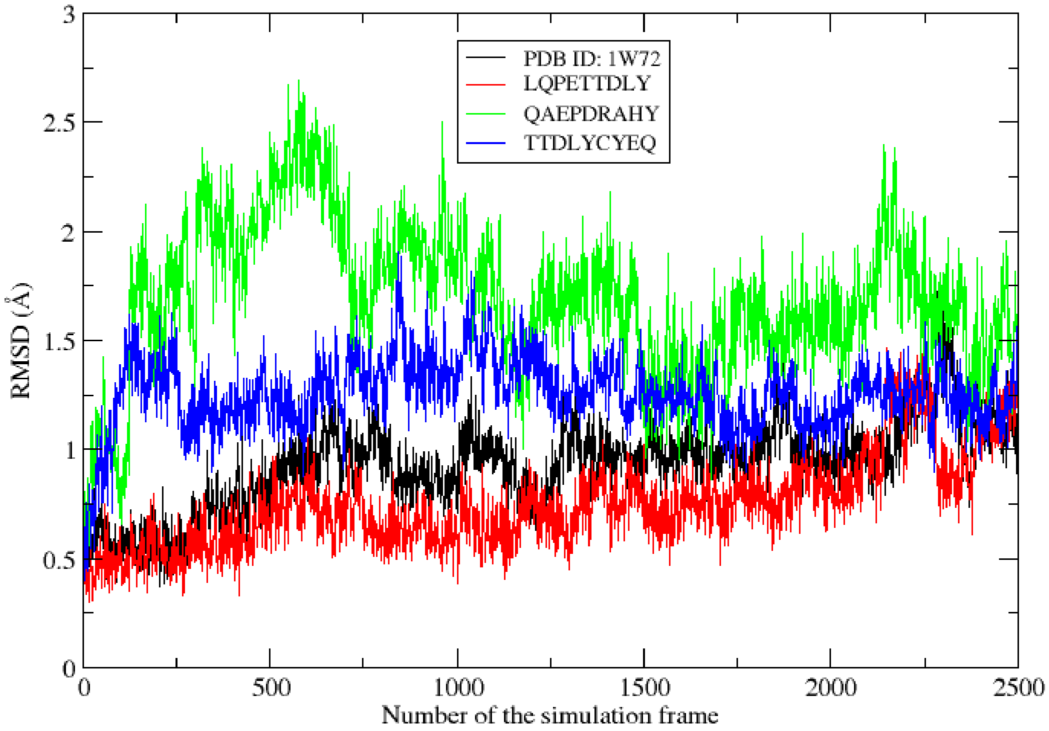

Fig. S19: RMSD of the backbone atoms of the peptide bound to HLA-A*01:01during the 10 ns MD simulation.

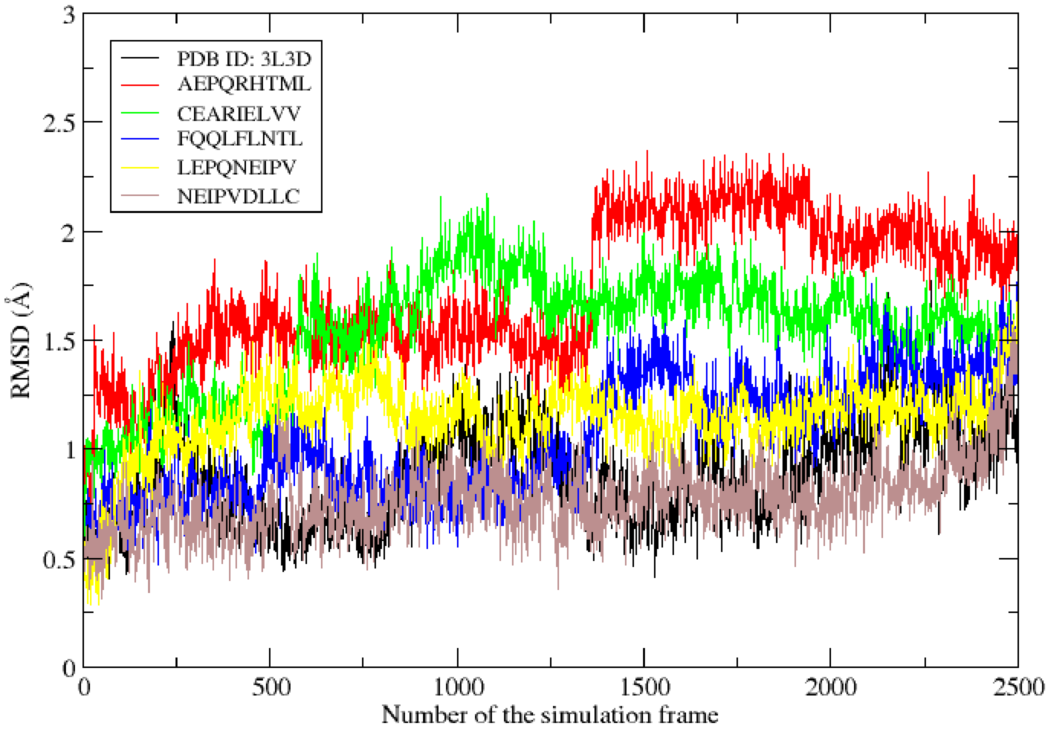

Fig. S20: RMSD of the backbone atoms of the peptide bound to HLA-B*44:02during the 10 ns MD simulation

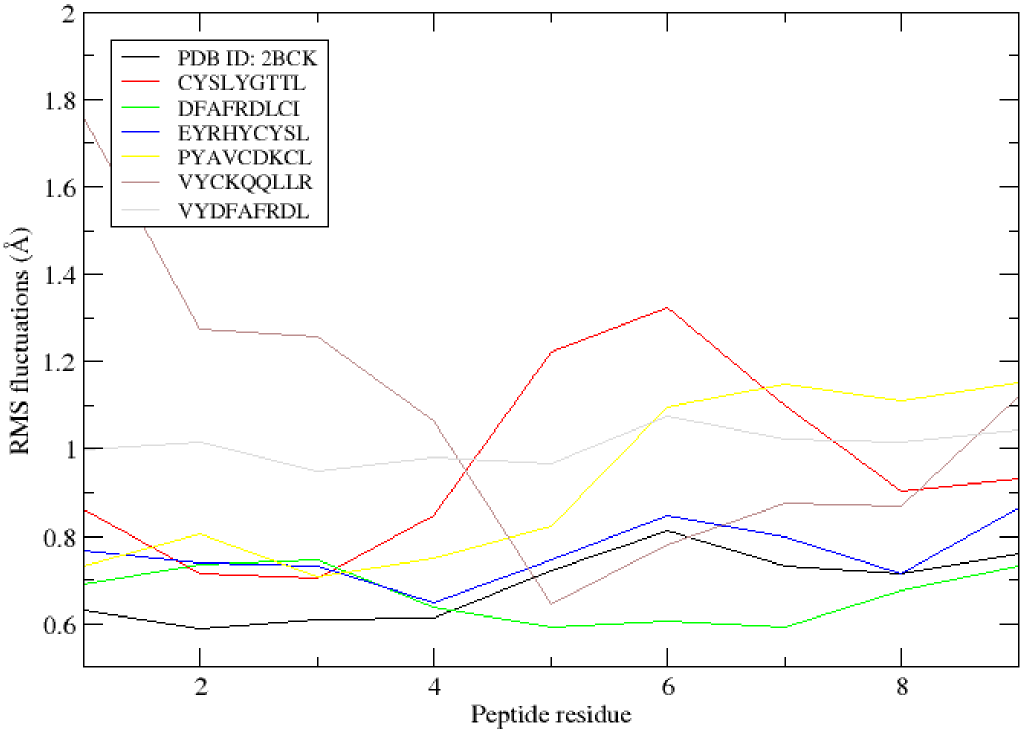

Figure S21: Per-residue RMS fluctuations of the E6 viral protein peptide from HPV type 16 in the binding groove of MHC-I HLA-A*24:02 during the last 4 ns of the MD simulations.

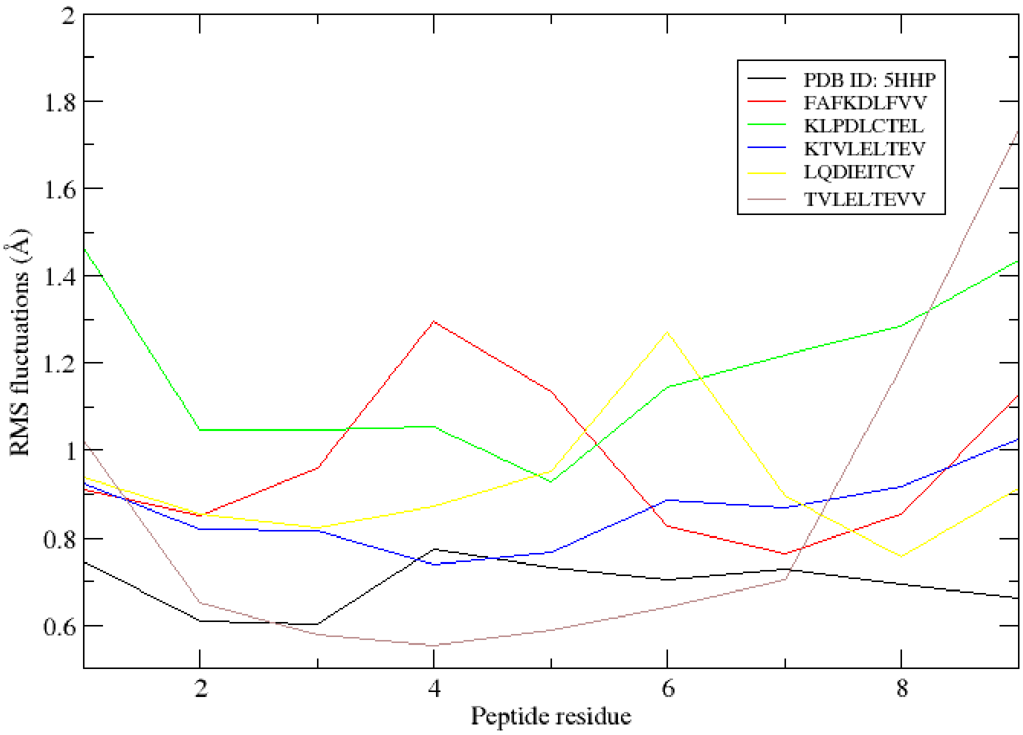

Fig. S22: Per-residue RMS fluctuations of the E6 viral protein peptide from HPV type 18 in the binding groove of MHC-I HLA-A*02:01 during the last 4 ns of the MD simulations.

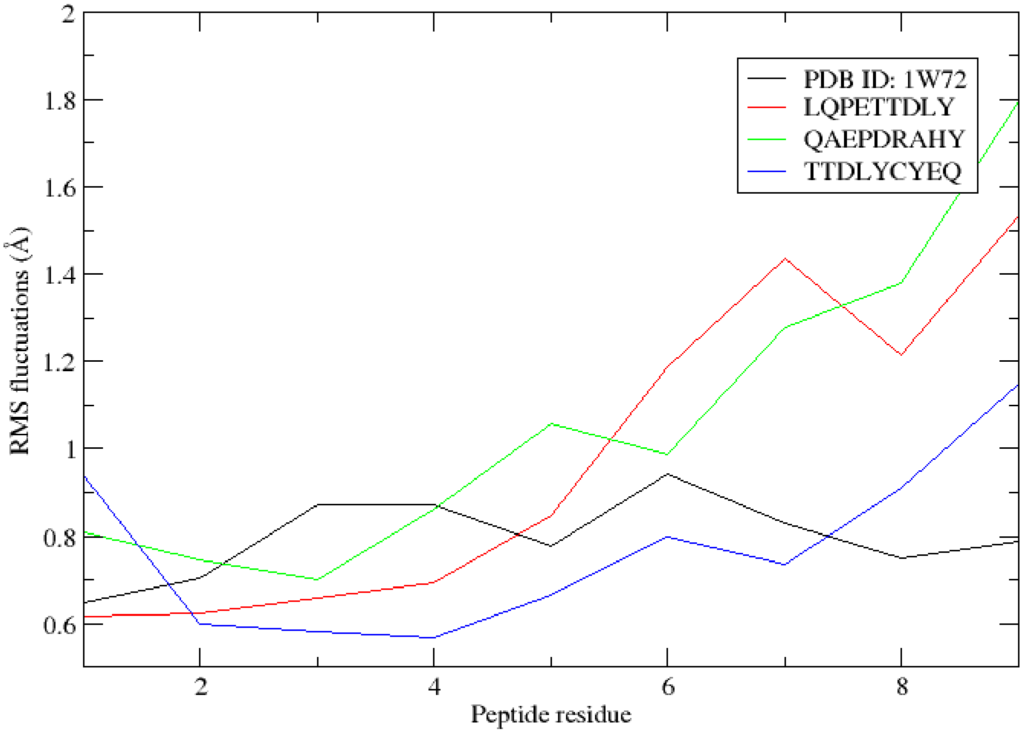

Fig. S23: Per-residue RMS fluctuations of the E7 viral protein peptide from HPV type 16 in the binding groove of MHC-I HLA-A*01:01 during the last 4 ns of the MD simulations.

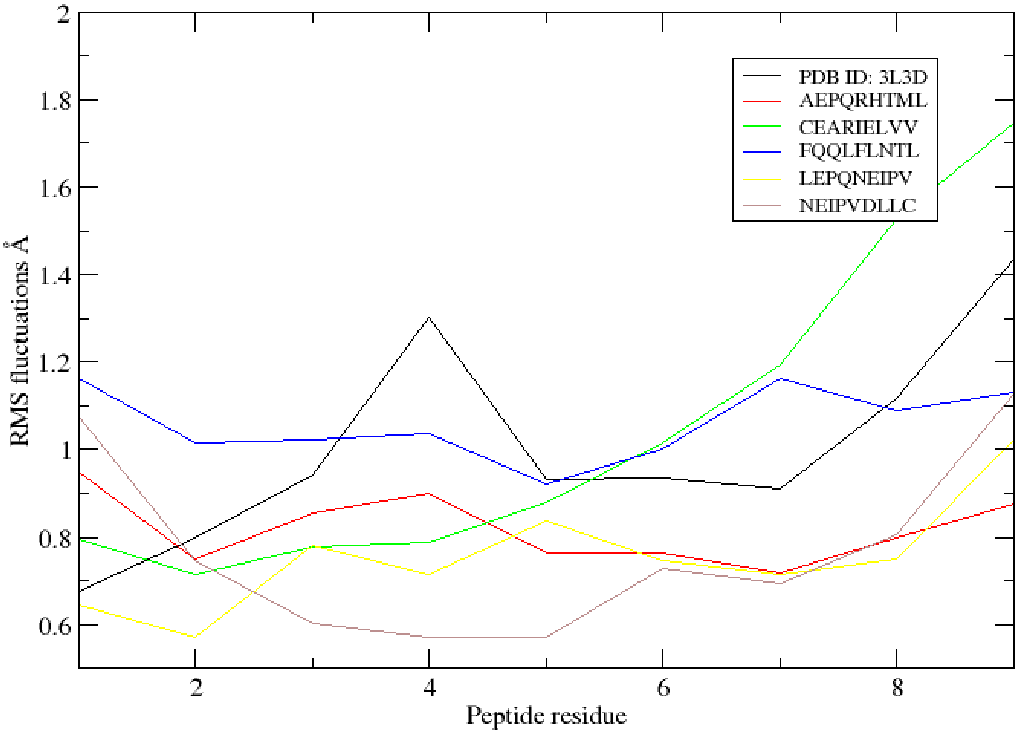

Fig. S24: Per-residue RMS fluctuations of the E7 viral protein peptide from HPV type 18 in the binding groove of MHC-I HLA-B*44:02 during the last 4 ns of the MD simulations.

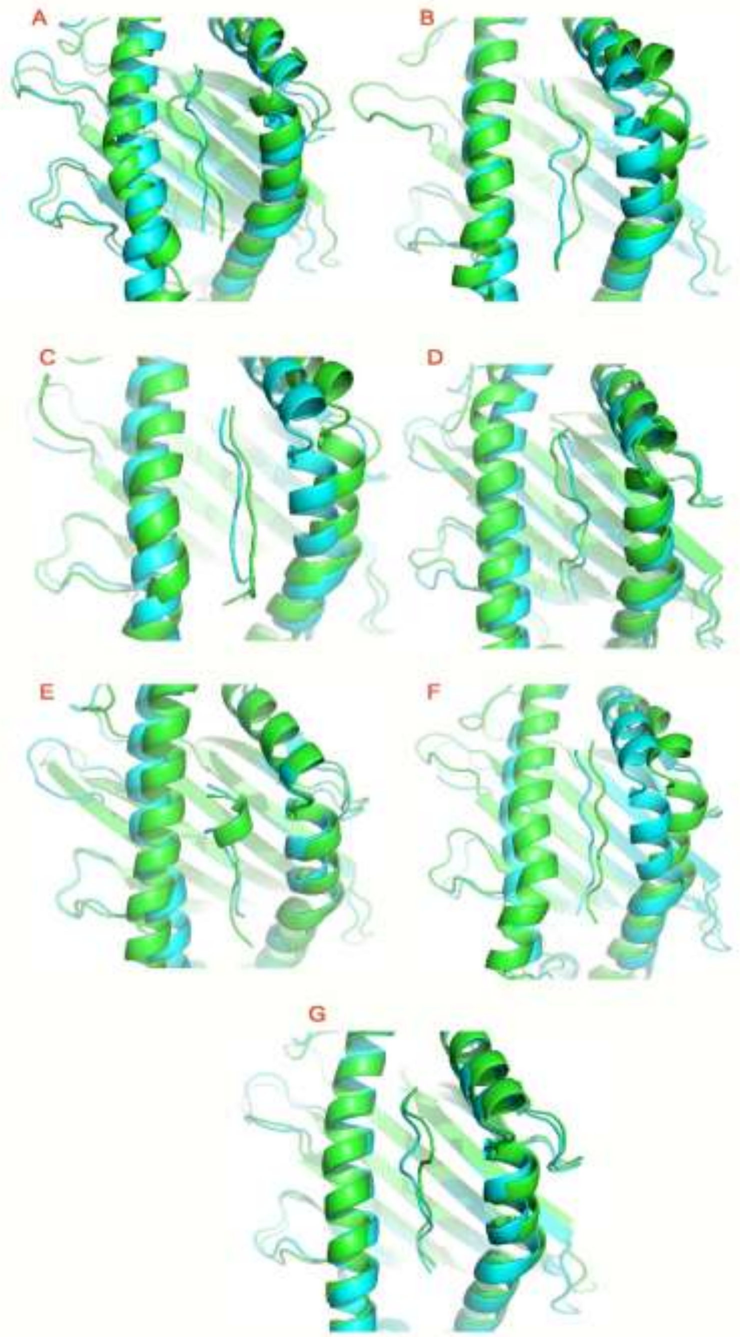

Fig. S25: (A-F) E6 viral protein peptides from HPV type 16 in the binding groove of MHC-I HLA-A*24:02; (G) Crystal complex of structure of HLA-A*24:02 allele with a telomerase peptide (PDB ID: 2BCK) (cartoon representation); the complex obtained from docking or Protein Data Bank (in cyan) is superimposed with the complex obtained after 10-ns MD simulation and subsequent energy minimization (in green): (A) CYSLYGTTL; (B) DFAFRDLCI; (C) EYRHYCYSL; (D) PYAVCDKCL; (E) VYCKQQLLR; (F) VYDFAFRDL; (G) VYGFVRACL (2BCK).

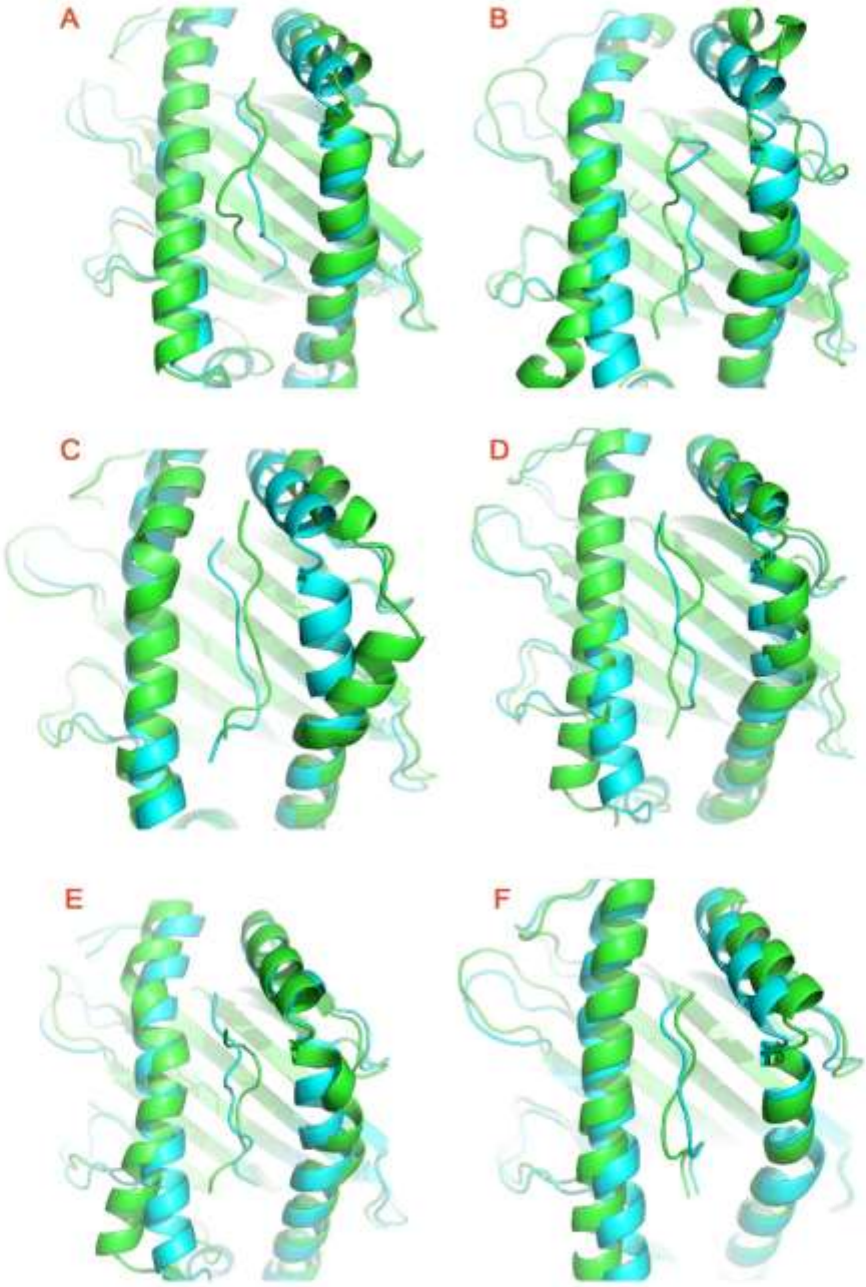

Fig. S26: (A-E) E6 viral protein peptides from HPV type 18 in the binding groove of MHC-I HLA-A*02:01 (PDB ID: 3HLA); (F) Crystal complex of structure of HLA-A*02:01 allele influenza A virus epitope variant M1-G4E (PDB ID: 5HHP) (cartoon representation); the initial complex obtained from docking or Protein Data Bank (in cyan) is superimposed with the complex obtained after 10-ns MD simulation and subsequent energy minimization (in green): (A) FAFKDLFVV; (B) KLPDLCTEL; (C) KTVLELTEV; (D) LQDIEITCV; (E) TVLELTEVV; (F) GILEFVFTL (5HHP).

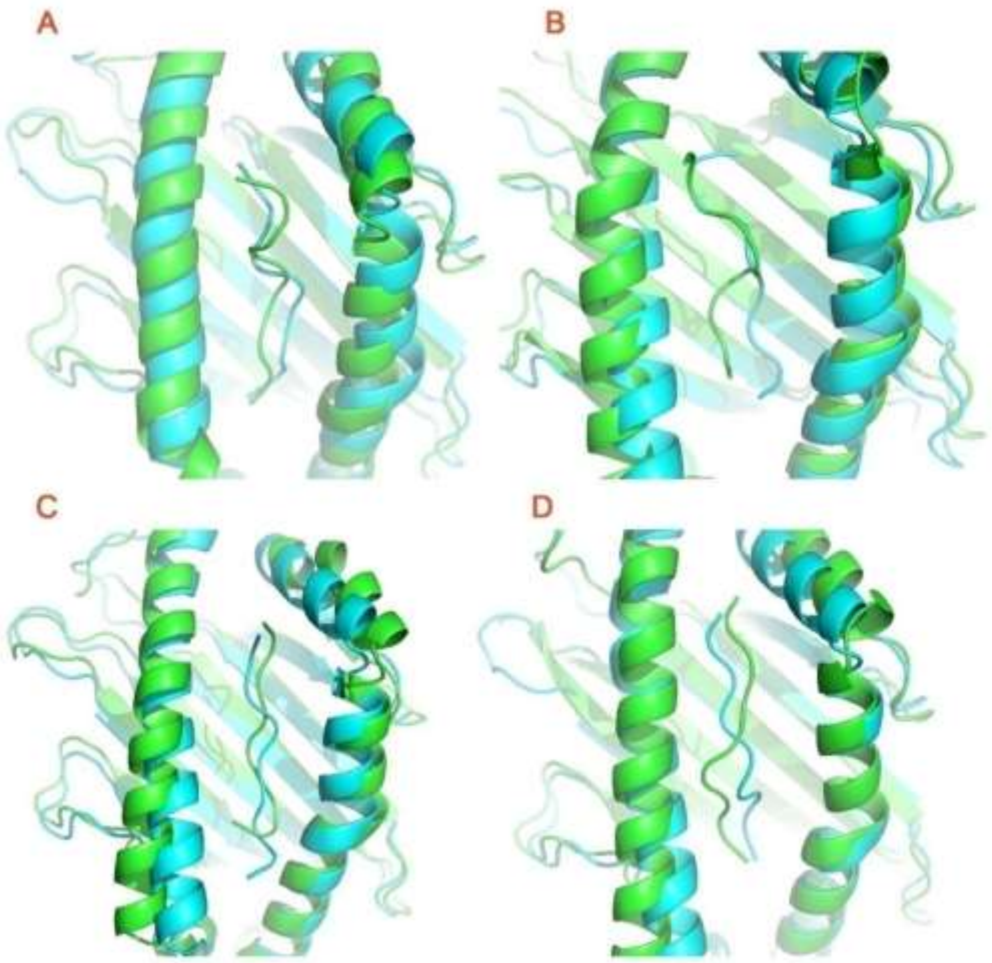

Fig. S27: (A-C) E7 viral protein peptides from HPV type 16 in the binding groove of MHC-I HLA-A*01:01; (D) Crystal complex of structure of HLA-A*01:01 allele with melanoma-associated antigen 1(PDB ID: 1W72) (cartoon representation); the initial complex obtained from docking or Protein Data Bank (in cyan) is superimposed with the complex obtained after 10-ns MD simulation and subsequent energy minimization (in green): (A) LQPETTDLY; (B) QAEPDRAHY; (C) TTDLYCYEQ; (D) EADPTGHSY (1W72).

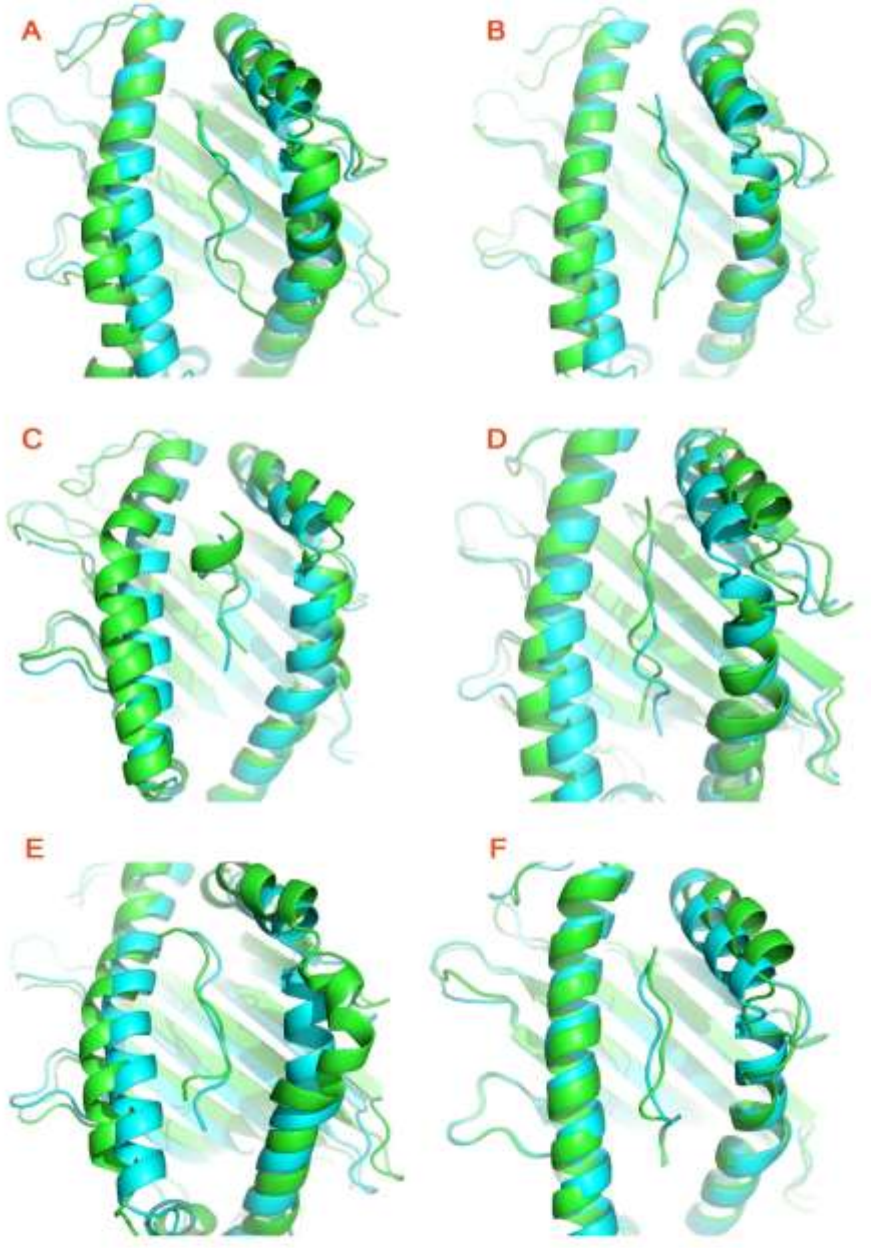

Fig. S28: (A-E) E7 viral protein peptides from HPV type 18 in the binding groove of MHC-I HLA-B*44:02; (F) Crystal complex of structure of HLA-B*44:02 allele with F3A mutant of a self-peptide derived from DPA*0201 (PDB ID: 3L3D) (cartoon representation); the initial complex obtained from docking or Protein Data Bank (in cyan) is superimposed with the complex obtained after 10-ns MD simulation and subsequent energy minimization (in green): (A) AEPQRHTML; (B) CEARIELVV; (C) FQQLFLNTL; (D) LEPQNEIPV; (E) NEIPVDLLC; (F) EEAGRAFSF (3L3D).
